# Common variants in *ZMIZ1* and near *NGF* confer risk for primary dysmenorrhoea

**DOI:** 10.1038/ncomms14900

**Published:** 2017-04-27

**Authors:** Zhiqiang Li, Jianhua Chen, Ying Zhao, Yujiong Wang, Jinrui Xu, Jue Ji, Jingyi Shen, Weiping Zhang, Zuosong Chen, Qilin Sun, Lijuan Mao, Shulin Cheng, Bo Yang, Dongtao Zhang, Yufeng Xu, Yingying Zhao, Danping Liu, Yinhuan Shen, Weijie Zhang, Changgui Li, Jiawei Shen, Yongyong Shi

**Affiliations:** 1 The Affiliated Hospital of Qingdao University & The Biomedical Sciences Institute of Qingdao University (Qingdao Branch of SJTU Bio-X Institutes), Qingdao University, Qingdao 266003, China; 2 Bio-X Institutes, Key Laboratory for the Genetics of Developmental and Neuropsychiatric Disorders (Ministry of Education), the Collaborative Innovation Center for Brain Science, Shanghai Jiao Tong University, Shanghai 200030, China; 3 Institute of Social Cognitive and Behavioral Sciences, Shanghai Jiao Tong University, Shanghai 200240, China; 4 Institute of Neuropsychiatric Science and Systems Biological Medicine, Shanghai Jiao Tong University, Shanghai 200042, China; 5 Shanghai Key Laboratory of Psychotic Disorders, Shanghai Mental Health Center, Shanghai Jiao Tong University School of Medicine, Shanghai 200030, China; 6 Physical Education Department, Shanghai Jiao Tong University, Shanghai 200042, China; 7 Key Laboratory of Ministry of Education for Conservation and Utilization of Special Biological Resources in the Western China, Ningxia University, Yinchuan 750021, China; 8 Campus Health Center of Shanghai Jiao Tong University, Shanghai 200042, China; 9 School of Physical Education and Coaching, Shanghai University of Sport, Shanghai 200438, China; 10 Shandong Provincial Key Laboratory of Metabolic Disease & the Metabolic Disease Institute of Qingdao University, Qingdao 266003, China; 11 Changning Mental Health Center, Shanghai 200042, China

## Abstract

Primary dysmenorrhoea, defined as painful menstrual cramps in the absence of pelvic pathology, is a common problem in women of reproductive age. Its aetiology and pathophysiology remain largely unknown. Here we performed a two-stage genome-wide association study and subsequent replication study to identify genetic factors associated with primary dysmenorrhoea in a total of 6,770 Chinese individuals. Our analysis provided evidence of a significant (*P*<5 × 10^−8^) association at rs76518691 in the gene *ZMIZ1* and at rs7523831 near *NGF*. *ZMIZ1* has previously been associated with several autoimmune diseases, and *NGF* plays a key role in the generation of pain and hyperalgesia and has been associated with migraine. These findings provide future directions for research on susceptibility mechanisms for primary dysmenorrhoea. Furthermore, our genetic architecture analysis provides molecular support for the heritability and polygenic nature of this condition.

## Abstract (web summary)

Primary dysmenorrhoea, the most common gynaecologic complaint, remains genetically and pathophysiologically elusive. Here, Li and colleagues identify common variants in *ZMIZ1* and near *NGF* conferring risk for primary dysmenorrhoea using genome-wide association study in a Chinese population.

Primary dysmenorrhoea is a medical condition characterized by menstrual pain without any evident pelvic pathology^1^. It causes significant disruption in quality of life and can result in absenteeism. It is thought to be the most common gynaecologic complaint among women, with a prevalence estimated at nearly 50% in menstruating females^2^. The causes of primary dysmenorrhoea are not precisely understood. Both emotional or psychological and physiological factors have been considered causes of primary dysmenorrhoea^3^, with anxiety^4^, stress^5^ and prostaglandin production^6^,^7^ reported as contributing factors. Twin studies suggest that primary dysmenorrhoea is heritable^8^,^9^,^10^. However, very few specific genetic variants have been found to be associated with primary dysmenorrhoea^11^,^12^, and these studies had small sample sizes. Thus, the genetic cause and mechanism of primary dysmenorrhoea are largely unknown. The discovery of genetic variants associated with primary dysmenorrhoea may lead to insights regarding its underlying biological mechanisms and could facilitate management of the condition (including the selection of medical and relief treatments).

Here we performed a two-stage genome-wide association study and subsequent replication study of primary dysmenorrhoea using 6,770 Chinese individuals. We identified two susceptibility loci in *ZMIZ1* and near *NGF* for primary dysmenorrhoea, and provided further genetic evidence for the polygenic nature of primary dysmenorrhoea. Our study provides new insights into the genetic architecture of primary dysmenorrhoea and suggests directions for future research.

## Results

### Primary dysmenorrhoea GWAS susceptibility loci

**Figure 1:**
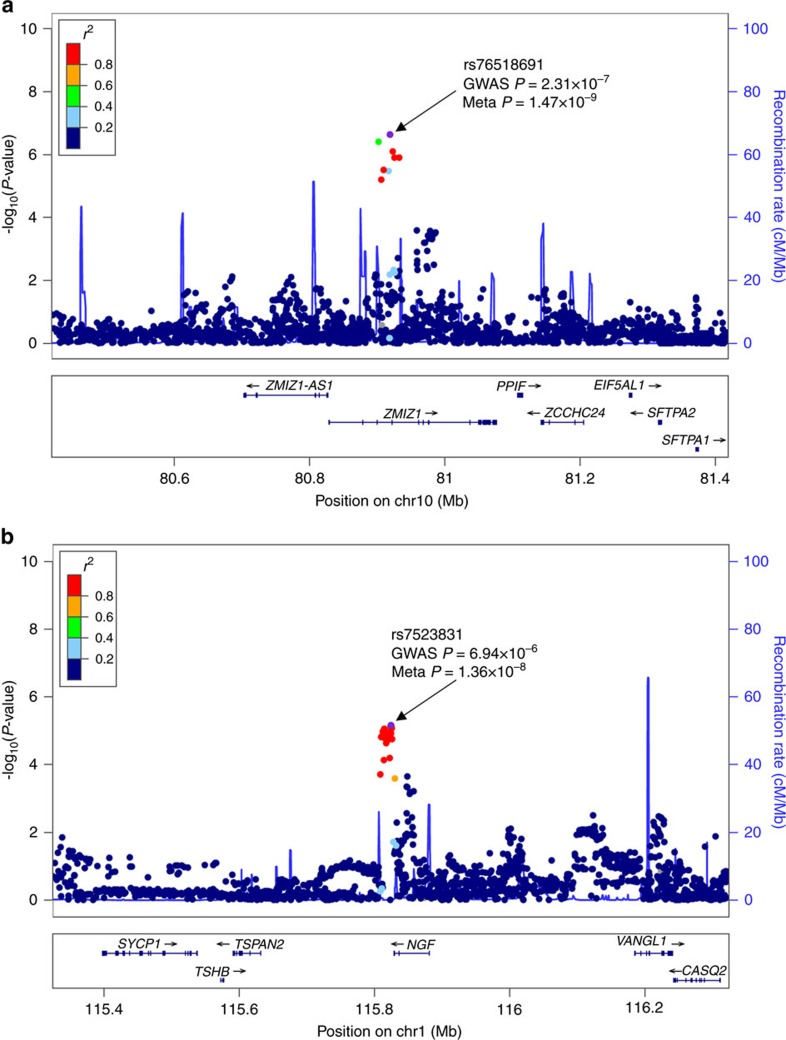
Regional association plots of loci associated with primary dysmenorrhoea. (**a**) rs76518691 and (**b**) rs7523831. Purple circles represent the most significantly associated SNP (marker SNP) in each region in the meta-analysis of discovery and replication. −log10 *P* values (*y* axis) of the SNPs (within the regions spanning 500 kb on either side of the marker SNP) are presented according to the chromosomal positions of the SNPs (*x* axis, hg19). SNPs are coloured according to their linkage disequilibrium (LD) with the marker SNP. The LD values were established based on the 1000 Genome Asian (ASI) data (March 2012). Estimated recombination rates with samples from the 1000 Genomes Project March 2012 release are shown as blue lines, and the genomic locations of genes within the interested regions annotated from the UCSC Genome Browser are shown as arrows.

**Figure 2:**
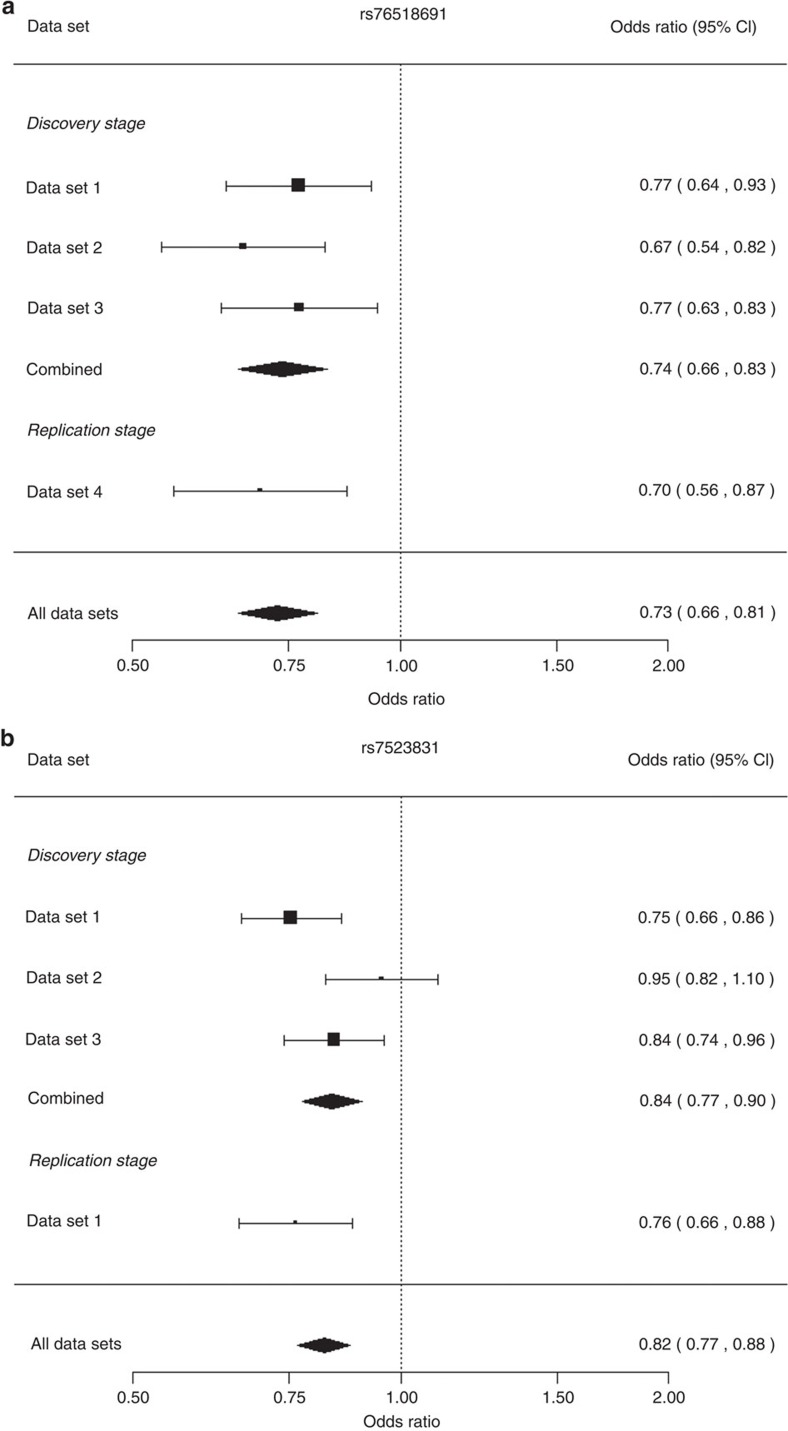
Forest plots for the two genome-wide significant SNPs. rs76518691 (**a**) and rs7523831 (**b**). The plots show the subset-specific odds ratios and 95% confidence intervals for all data sets in the discovery and replication stages presented as rectangles and bars, respectively. For each SNP, the association estimate and confidence interval for the fixed-effect meta-analysis combining discovery stage and all data sets results are presented as a diamond.

In the discovery phase, a genome-wide association study (GWAS) meta-analysis was performed across three cohorts comprising a total of 5,324 individuals (2,404 cases and 2,920 controls; Supplementary Tables 1 and 2). Principal component analysis (PCA) was applied to identify population substructures within the samples (Supplementary Fig. 1). To enable integrative and cross-platform analysis, genotype imputations were employed in all studies using the reference data from the 1000 Genomes Project March 2012 release. Association analyses with adjustments for potential population stratification were performed at the cohort level. The results were combined using inverse-variance meta-analysis based on the fixed-effect model. A total of 6,277,361 single-nucleotide polymorphisms (SNPs) passed quality control and were kept for further analysis. A genomic inflation factor of 1.018 was observed, suggesting that the confounding effects of population stratification were well controlled (Supplementary Fig. 2). From the discovery stage, no genome-wide significant (GWS) locus (*P*<5 × 10^−8^) was identified (Supplementary Fig. 3). We then took the SNP with the lowest *P* value among the 16 loci with *P*<1 × 10^−5^ for replication in an independent cohort of 1,446 individuals (678 cases and 768 controls, Supplementary Tables 1 and 3). In a subsequent meta-analysis of the discovery and replication samples, we identified two GWS SNPs: rs76518691 (*P*=1.47 × 10^−9^), an intron variant in *ZMIZ1*, and rs7523831 (*P*=1.36 × 10^−8^), located close to *NGF* (Table 1, Figs 1 and 2). For both of the GWS SNPs, there was no evidence to suggest significant heterogeneity across discovery and replications (*P*_het_>0.05, Supplementary Table 3).

### Functional implications of the susceptibility loci

To explore the potential implications and epigenetic profile of the association signals, we queried annotations of the non-coding genome for the index SNPs and those in linkage disequilibrium (LD) with them (*r*^2^>0.8, based on the 1000 Genomes Project ASN data set). Many of these SNPs, including the significant SNPs rs76518691 and rs7523831, are predicted to reside within promoter and/or enhancer elements, suggesting putative regulatory functionality for these loci (Supplementary Table 4). Further tissue-specific regulatory analysis using the Roadmap Epigenome and ENCODE integrated data sets highlighted several tissues and cell types, including adipose-derived mesenchymal stem cells (AD-MSC; H3K4me3 and H3K4me1 signals at the region surrounding rs76518691 and H3K4me3 signal at the region surrounding rs7523831), bone marrow-derived mesenchymal stem cell (BD-MSC) culture cells (H3K4me3 and H3K27ac signals for rs7523831), cortex and ganglionic eminence-derived neurospheres (H3K4me1 signal for rs7523831) and some muscle-related tissues (Supplementary Figs 4 to 9). Some of these cell types might be biologically relevant. For instance, human AD-MSCs have been shown to promote neurogenesis^13^ and to play a neuroprotective role^14^ in animal studies. In addition, human BM-MSCs and AD-MSCs were found to downregulate inflammatory and T-cell responses *in vitro*^15^,^16^. Both the cortex and ganglionic eminence are regions of the nervous system where pain is mediated through complicated processing pathways^17^.

To assist in interpreting and contextualizing the results, we investigated whether the loci associated with primary dysmenorrhoea were previously suspected of being involved in the aetiology of other diseases or traits and conducted functional annotation searches of published literature. We reasoned that any prior knowledge might help further unravel the potential roles of the loci. Variants in or near *ZMIZ1* have been found to be associated with several complex diseases or traits (Supplementary Table 5), including multiple autoimmune diseases (that is, inflammatory bowel disease, multiple sclerosis, celiac disease, vitiligo, Crohn's disease and psoriasis). *ZMIZ1* encodes the zinc finger protein MIZ type 1, which regulates TGF-beta/SMAD signalling^18^ to play critical roles in the immune system^19^. A variant near *NGF* is associated with migraine (Supplementary Table 5), a major headache disorder. Most recently, a GWAS in female individuals of European descent identified *NGF* as pain-severity-associated locus in dysmenorrhoea (regardless of primary or secondary types)^20^. *NGF* encodes a secreted protein that possesses nerve growth stimulating activity and is critical for the survival and maintenance of sympathetic and sensory neurons. In addition, NGF exerts biological effects on a variety of immune system cells^21^ and contributes to the immune response^22^ and is involved in a number of autoimmune disorders such as rheumatoid arthritis^23^ and psoriasis^24^. Moreover, prostaglandins, physiological factors probably involved in primary dysmenorrhoea, are thought to be powerful inducers of NGF^25^. Under inflammatory conditions, the NGF level increases and induces axonal outgrowth in nearby pain neurons, which leads to increased pain perception^26^.

### Genome-wide gene and pathway analysis

To leverage our genome-wide data set and interpret the results, we conducted complementary gene-based and pathway-based association analyses. In the gene-based analysis, none of the tested genes reached significance after Bonferroni correction (*P*<2 × 10^−6^), and the most associated gene was *IL1A* (*P*=2.60 × 10^−5^, Supplementary Table 6). *IL1A* encodes a pleiotropic cytokine that is a member of the interleukin-1 cytokine family. This cytokine is involved in various inflammatory processes and hematopoiesis and plays one of the most important roles in the regulation of the immune response. We also identified five gene sets that were enriched at a false discovery rate <0.05, the top three of which included interleukin-1 receptor binding, the amylase family and the bone morphogenic protein (BMP) signalling pathway (Supplementary Table 7).

### SNP heritability of primary dysmenorrhoea

To determine the proportion of variation in susceptibility to primary dysmenorrhoea that is captured by common variants, we used the restricted maximum likelihood method to estimate the total variance explained by the genotyped and imputed SNPs (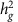) using the discovery cohort data sets. We observed an 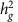 of 22.9% (s.e.=5.8%) from the autosomal SNPs. We also sought to partition the heritability across functional categories to assess enrichment. Of the six primary functional annotation categories proposed by Gusev *et al*.^27^, the highest enrichment was observed for coding variants, which explained 24.0% (s.e.=17.8%) of the 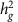 within 1.5% of SNPs (16.3 × enrichment). DNaseI hypersensitivity sites (DHSs) spanned 16.1% of SNPs but explained approximately 60.9% (s.e.=46.7%) of the 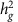 (3.8 × enrichment), and no enrichment was observed for the other four categories (Supplementary Table 8).

### Effects of endometriosis variants on primary dysmenorrhoea

Endometriosis is the most common cause of secondary dysmenorrhoea. Several GWASs have been conducted on patients with endometriosis, and six GWS loci have been identified (Supplementary Table 9). We investigated whether the previously identified endometriosis risk alleles also confer risk to primary dysmenorrhoea. Of the seven GWS SNPs, one (rs1537377) showed nominal significant association but in the opposite direction of our GWAS discovery samples (Supplementary Table 10). However, we also noted that a SNP (rs6542095) near *IL1A* (the most significantly associated gene from our gene-based analysis) was found to be associated near GWS with endometriosis (*P*=3.45 × 10^−7^) and at GWS with moderate-to-severe endometriosis (*P*=3.43 × 10^−8^)^28^. We observed association between rs6542095 and primary dysmenorrhoea with a *P* value of 1.71 × 10^−4^, and the risk allele showed directional consistency across diseases.

## Discussion

Primary dysmenorrhoea, which affects >50% of menstruating women, causes extensive personal and public health problems, yet knowledge of its pathophysiology and aetiology is limited. Genome-wide association analysis is a hypothesis-free approach that has been widely and successfully applied in genetic analysis of complex traits and diseases^29^. In this study, we adopted a two-stage design analysis: we followed the initial genome-wide genotyping of 2,404 cases and 2338 controls by testing the top most associated SNPs from the discovery stage in an independent replication cohort of 678 cases and 768 controls. We identified two GWS association signals at rs76518691 (in *ZMIZ1*) and rs7523831 (near *NGF*) in the joint analysis of the discovery and replication cohorts. We also conducted supplemental analyses to aid in interpreting the results. Functional annotation suggested potential regulatory functionality for both loci, and cell-type specific analysis highlighted chromatin mark (for example, H3K4me3, H3K4me1 and so on) signals at some possible biologically relevant tissues, including AD-MSCs, BM-MSCs and the cortex and ganglionic eminence. *ZMIZ1* has previously been associated with multiple autoimmune diseases. *NGF* has been shown to play a critical role in the generation of pain and has been associated with migraine and pain severity in dysmenorrhoea. *NGF* is also known to be involved in some autoimmune disorders. Previous researches demonstrated obvious immunological functions for both *ZMIZ1* and *NGF*, suggesting a potential role for the immune system in the pathogenesis of primary dysmenorrhoea.

The gene-based and pathway-based analyses also supported a role of the immune system in primary dysmenorrhoea. In the gene-based analysis, the top significant gene is *IL1A*, which encodes a key regulator of immune and inflammatory processes. The pathway analysis highlighted several gene sets, including interleukin-1 receptor binding and the BMP signalling pathway. Interleukin-1 receptor binding plays an important role in mediating many immune and inflammatory responses^30^, while the BMP signalling pathway is thought to have important roles in the regulation of cell survival, proliferation and differentiation in the immune system^31^. It should be mentioned that *IL1A* has been robustly implicated in endometriosis^28^,^32^, which is the most common cause of secondary dysmenorrhoea. However, we did not observe any other clear evidence for shared genetic backgrounds between endometriosis and primary dysmenorrhoea in the previously reported GWAS findings. Thus, further studies are required to fully clarify the relationship between these two diseases.

We estimated that ∼23% of the variance in primary dysmenorrhoea can be explained by the autosomal SNPs. Of them, the DHS and coding variants explained about 61% and 24% of 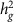, respectively. In the context of the GWAS literature, the estimated 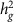 for primary dysmenorrhoea was comparable to the values observed for other complex traits and diseases, such as body mass index (∼16%)^33^ and schizophrenia (∼23%)^34^. For partitioned heritability for specific functional categories, enrichment was observed for the DHSs category (explaining over 50% of the estimated 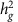), similar to other common diseases^27^. Our findings suggest that primary dysmenorrhoea is a highly polygenic condition.

There are some limitations in this study. In our two-stage GWAS with multiple data sets, the two highlighted SNPs reached GWS only after joint analysis of the discovery and replication cohorts, especially for rs7523831 with ‘Data set 2' producing a non-significant *P* value (>0.05). Nonetheless, the association signals for both GWS SNPs are consistent across at least four data sets, so that they are unlikely to be false positives. Therefore, we believe that the sample size in this study is still relatively small and the statistical power is limited. Moreover, on the clinical settings, the secondary dysmenorrhoea can only be ruled out by abdominal imaging examination. Thus, it is conceivable that the certain portion of the case group could be contaminated by the secondary aetiologies, which might impose some subtle influence on the final results.

In summary, we present the first GWAS for primary dysmenorrhoea in Chinese population and identify two GWS loci (*ZMIZ1* and *NGF*). The results provide further genetic evidence for the polygenic nature of primary dysmenorrhoea and show that a remarkable proportion of heritability can be captured by common SNPs. Our findings shed light on the genetic architecture of primary dysmenorrhoea and suggest future directions for research on susceptibility mechanisms. Additional GWASs involving multiple ethnic populations and functional studies are necessary to identify additional genetic factors and unravel the complex biology of primary dysmenorrhoea.

## Methods

### Study design

We performed a two-stage GWAS to identify loci associated with primary dysmenorrhoea in Chinese females. In the discovery stage, we performed a meta-analysis of three GWAS data sets (2,404 cases and 2,920 controls in total). The top significant variants were further tested in an independent data set (678 cases and 768 controls). All participants provided written informed consent. Approval was obtained from the Ethics Committee of Human Genetic Resources in Bio-X Institutes at Shanghai Jiao Tong University. We confirm that our study is compliant with the ‘Guidance of the Ministry of Science and Technology for the Review and Approval of Human Genetic Resources'. The descriptive statistics of the samples are provided in Supplementary Table 1.

### Phenotype

Dysmenorrhoea (or painful menstruation, MeSH ID: D004412) is a medical condition involving menstrual pain. Dysmenorrhoea is commonly divided into primary and secondary dysmenorrhoea on the basis of pathophysiology. Secondary dysmenorrhoea is associated with an identifiable disease (such as endometriosis), whereas primary dysmenorrhoea occurs without pelvic pathology^1^. In adolescents and young adults, the most prevalent type is primary dysmenorrhoea. A medical history and physical examination are usually sufficient to make a diagnosis of primary dysmenorrhoea^3^. We investigated ∼10,000 female students at Shanghai Jiao Tong University and Ningxia University by routine physical examination during a period spanning 2008–2014, and most of the participants were born over a period of 17 years (from 1980 to 1996) and ranging from 18 to 34 years of age at the time of sampling. A visual analogue scale has previously been used to rate menstrual pain^35^,^36^. In this study, we adopted a horizontal 10-cm visual analogue scale with endpoints spanning from ‘no pain at all' (score=0) on the far left to ‘the worst pain' (score=10) on the far right. Scores of less than 1 were assigned to a control group, and scores higher than 4 (moderate intensity)^36^ were assigned to the case group. The participants with possible causes of secondary dysmenorrhoea, such as endometriosis and other gynaecological problems^7^, were excluded. A total of 6,770 individuals were genotyped and analysed in this GWAS.

### Genotyping and quality control (QC)

For data set 1, genotyping was performed using Illumina HumanOmni1-Quad BeadChips according to the manufacturer's instructions (Illumina, Inc., San Diego, CA, USA). Genotype calls were generated using Genome Studio software. For data sets 2 and 3, genotyping was performed using the Affymetrix Axiom Genome-Wide CHB1 & CHB2 Array Plate Set according to the manufacturer's protocols (Affymetrix, Inc., Santa Clara, CA, USA). Genotype calls for the CHB1 and CHB2 arrays were generated separately according to the Axiom Genotyping Solution Data Analysis Guide. For data set 4, genotyping was performed using the Sequenom MassARRAY iPLEX platform following the manufacturer's instructions (Sequenom, Inc., San Diego, CA, USA). Genotype calls were made using SEQUENOM's Typer 4.0 software.

Systematic QC analysis at both the sample and SNP levels was performed separately for each GWAS data set. We first filtered out low-quality samples: samples with a call rate <97% in data set 1, and samples with a dish QC value <0.82 in data sets 2 and 3. Next, gender was estimated via genotyping data, and samples with inconsistent gender were removed. Heterozygosity rates were calculated for each sample, and deviations of more than 6 s.d. from the mean were excluded. PLINK's identity-by-descent (IBD) analysis was used to detect cryptic relatedness. The member (with a lower call rate) of the pair of unexpected duplicates or probable relatives (PI_HAT>0.20) was also excluded. For SNP QC, SNPs with call rates < 97%, MAF<3% or significant deviations from Hardy–Weinberg equilibrium (HWE) in the controls (HWE *P*≤1 × 10^−6^) were excluded. Subsequently, PCA was performed using a linkage disequilibrium (LD)-pruned SNP set to identify population substructures and outliers, which were removed. Detailed information regarding QC procedures is provided in Supplementary Table 2.

### Imputation

Ungenotyped variants were imputed to the post-quality control GWAS data using SHAPEIT 2.0 (phasing step)^37^ and IMPUTE2 (imputation step)^38^ software. The haplotype information was obtained from the 1000 Genomes Project (Phase I integrated variant set across all 1,092 individuals, v2, March 2012)^39^.

### Population stratification analysis

Population stratification was assessed using a PCA-based method implemented in the software package EIGENSTRAT^40^, and a total of 20 principal components were generated.

### Association and meta-analysis

SNPTEST^41^ was used for single SNP association using a case-control test assuming an additive genetic model. The first principal component from PCA analysis was included to control for unobserved population structure. For the replication study, allelic association analysis was conducted using SHEsis^42^,^43^. An inverse-variance method (fixed-effect model)-based meta-analysis was adopted to combine results from different data sets, and heterogeneity across the data sets was evaluated using Cochran's Q test, which was implemented in META^44^.

### Bioinformatic analysis and functional annotation of GWS loci

The genome-wide significant SNPs and variants in LD (*r*^2^>0.8 in the Asian 1000 Genomes Project population) were annotated using HaploReg^45^ and RegulomeDB^46^. These functional annotations include promoter and enhancer histone marks, DHSs, bound proteins, and altered motifs from the ENCODE^47^ and Roadmap Epigenomics Projects^48^, as well as GERP and SiPhy conservation scores. The Roadmap Epigenome Browser was used to explore the tissue-specific regulatory roles of genome-wide significant SNPs^49^. The integrated data from both the ENCODE and Roadmap Epigenomics Consortium Projects for histone profiles (H3K4me1, H3K4me3 and H3K27ac) of 46 primary human tissues and cells were analysed.

We extracted previously reported GWAS associations within 500 kb of the top significant SNPs from the NHGRI GWAS Catalog^50^, and we searched the published literature in PubMed for related genes to obtain additional functional evidence for these SNPs and genes.

### Gene- and pathway-based association analyses

We performed gene-based association testing using VEGAS2 (ref. 51) with the gene boundary option of ‘0kbloc' for SNP selection. We used MAGENTA^52^ to explore pathway-based associations in the GWAS meta-analysis data set (excluding the MHC region due to difficulties in accounting for the gene density and LD patterns^53^). An individual pathway that reached a false discovery rate *q*<0.05 in either analysis (95th and 75th percentiles cutoff) was considered to be suggestively enriched.

### Analysis of heritability

The proportion of variance to primary dysmenorrhoea explained by the common SNPs was estimated using a tool for genome-wide complex trait analysis (GCTA)^54^. For functional partitioning of SNP heritability, we used six primary categories (coding, UTR, promoter, DHSs, intronic and intergenic) that were proposed by Gusev *et al*.^27^. All six functional categories were jointly analysed.

### Data availability

Summary statistics of our genome-wide analysis can be downloaded from the Bio-X website (http://analysis.bio-x.cn/gwas/).

## Additional information

**How to cite this article:** Li, Z. *et al*. Common variants in *ZMIZ1* and near *NGF* confer risk for primary dysmenorrhoea. *Nat. Commun.* **8,** 14900 doi: 10.1038/ncomms14900 (2017).

**Publisher's note:** Springer Nature remains neutral with regard to jurisdictional claims in published maps and institutional affiliations.

## Supplementary Material

Supplementary Information Supplementary Figures and Supplementary Tables

## Figures and Tables

**Table 1:** Results for the two genome-wide significant SNPs.

**Info**	**Effect allele (Freq.)**	**Phase**	**OR (95% CI)**	***P*** **value**
rs7523831	C (0.543)	Data set 1	0.753 (0.662–0.857)	1.66 × 10^−5^
Chr1: 115,824,192		Data set 2	0.952 (0.822–1.102)	0.5074
*NGF*		Data set 3	0.842 (0.739–0.958)	9.27 × 10^−3^
Downstream		Discovery	0.837 (0.775–0.905)	6.94 × 10^−6^
		Data set 4	0.762 (0.659–0.882)	2.70 × 10^−4^
		**Combined**	**0.820 (0.766–0.878)**	**1.36 × 10^−8^**
				
rs76518691	A (0.133)	Data set 1	0.769 (0.638–0.927)	5.99 × 10^−3^
Chr10: 80,918,767		Data set 2	0.667 (0.539–0.824)	1.79 × 10^−4^
*ZMIZ1*		Data set 3	0.770 (0.629–0.943)	0.0114
Intron		Discovery	0.738 (0.657–0.828)	2.31 × 10^−7^
		Data set 4	0.696 (0.556–0.871)	1.55 × 10^−3^
		**Combined**	**0.729 (0.658–0.807)**	**1.47 × 10^−9^**

Freq., the frequency for the effect allele; OR, odd ratio; SNP, single-nucleotide polymorphism; 95% CI, 95% confidence interval. Info, information for SNP (SNP id, chromosome: position, gene and location). The chromosomal positions are based on hg19. Genome-wide significant (*P*<5 × 10^−8^) results are shown in bold.
