# Supplementary material for: Common variants in ZMIZ1 and near NGF confer risk for primary dysmenorrhoea
Source: Nat Commun. 2017 Apr 27;8:14900. doi: 10.1038/ncomms14900 (PMC5414039; doi:10.1038/ncomms14900)
Supplement: Supplementary Information — Supplementary Figures and Supplementary Tables [file ncomms14900-s1.pdf]

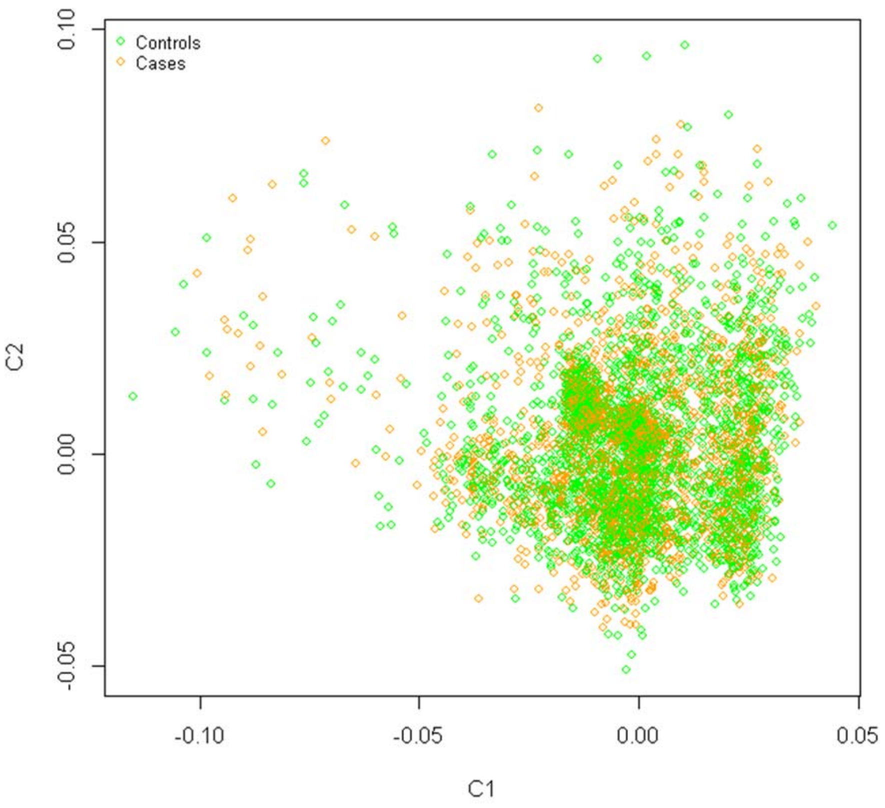

Supplementary Figure 1. Principal Components Analysis (PCA) of samples from the GWAS discovery stage. Cases and controls are projected onto the top two principal components of genetic stratification. Cases are represented by orange circles, whereas controls are in green.

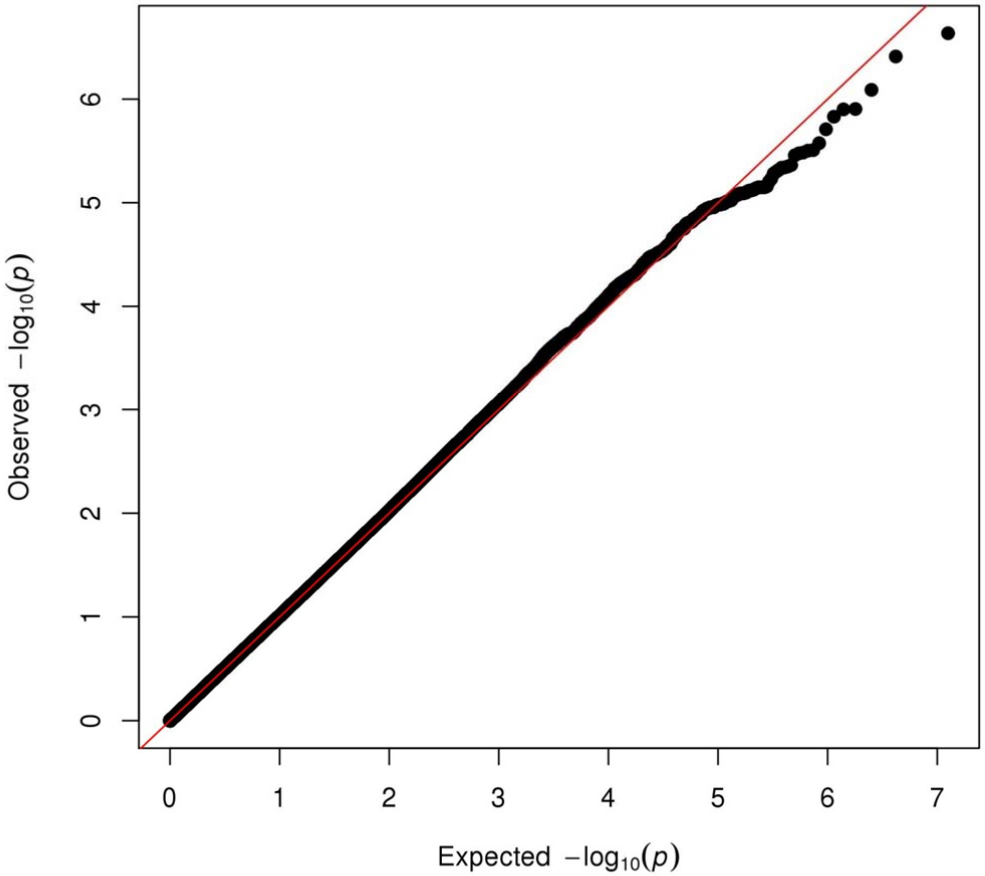

Supplementary Figure 2. Quantile-quantile (Q-Q) plot showing the association results for the GWAS discovery stage. The Q-Q plot representative of observed (y axis) vs. expected (x axis) SNPs distribution.

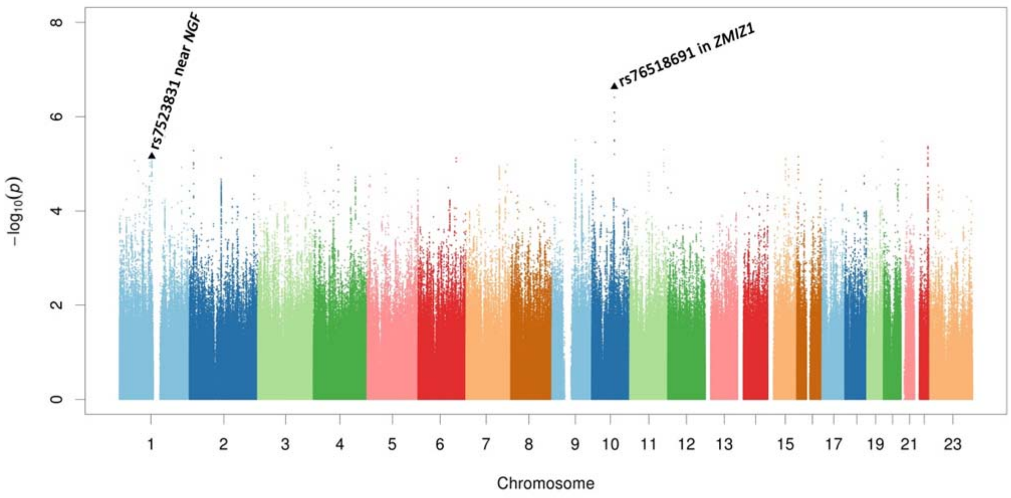

Supplementary Figure 3. Manhattan plot from the GWAS discovery stage meta-analysis. The Manhattan plot shows negative log10-transformed P values from meta-analysis over the whole genome. None genome-wide significant (GWS) SNP (P $< 5 \times 10^{-8}$) was observed in the discovery stage. Two SNPs (rs76518691 in *ZMIZ1* and rs7523831 near *NGF*, marked in black triangles) reached GWS in the meta-analysis of the discovery and replication samples.

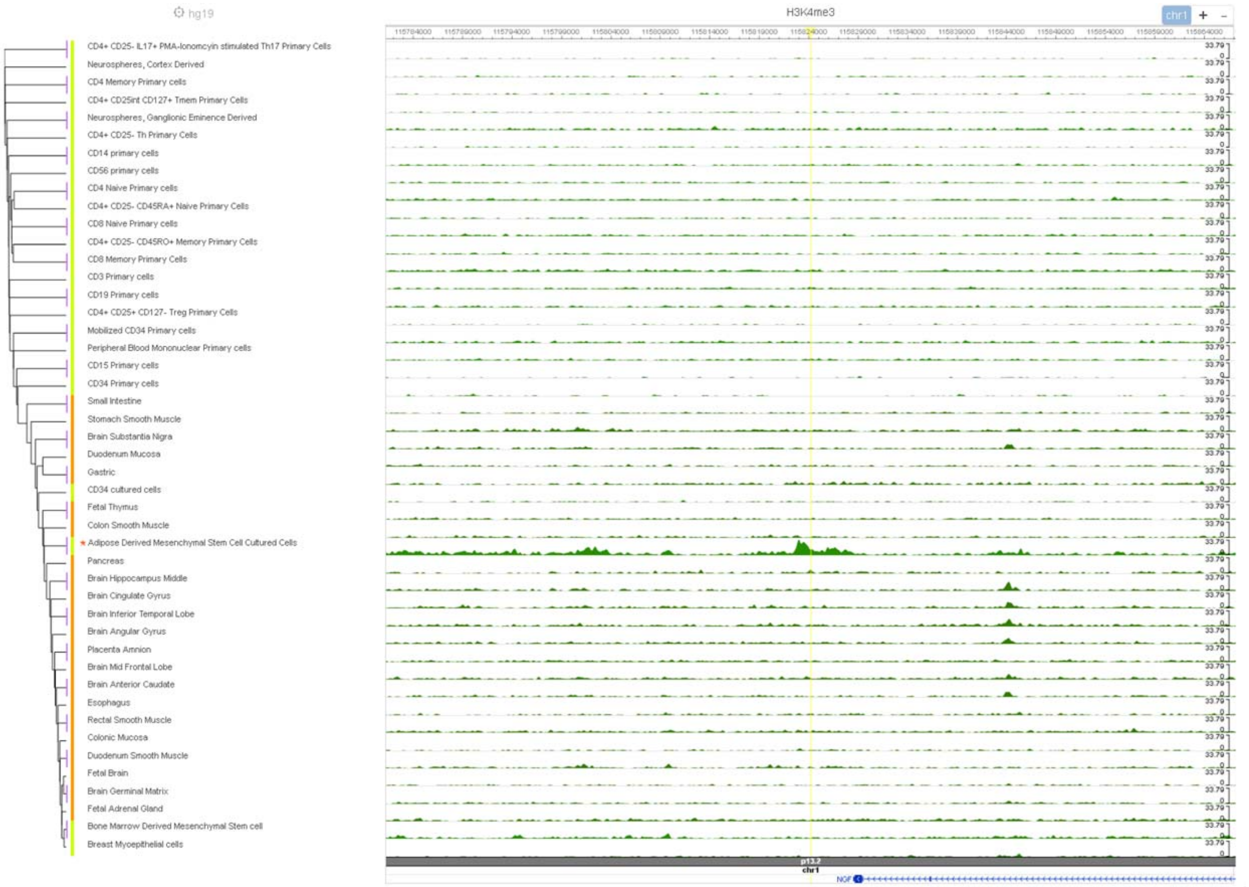

Supplementary Figure 4. Epigenomic annotation (H3K4me3) for the region surrounding rs7523831. The yellow vertical line indicates the position of the queried SNP.

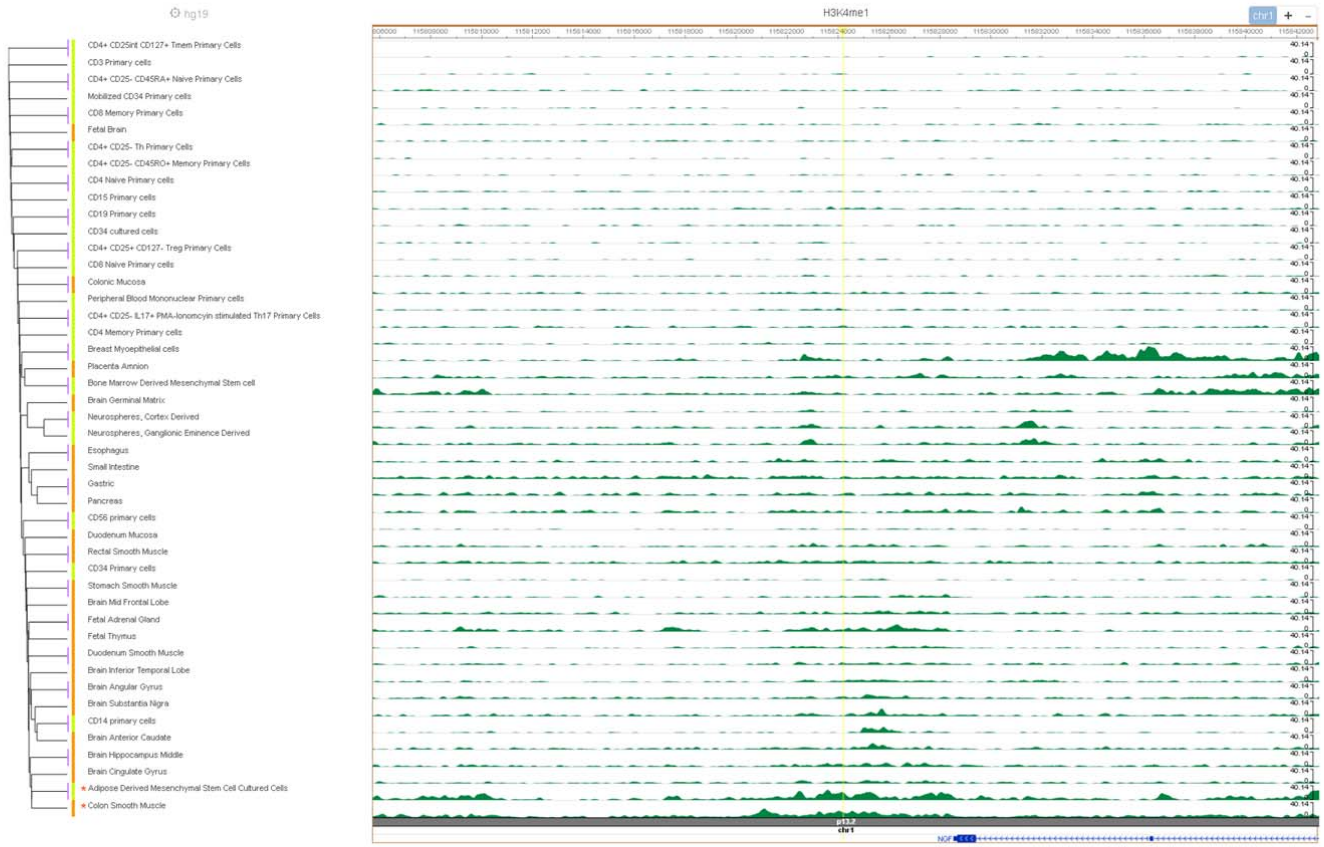

Supplementary Figure 5. Epigenomic annotation (H3K4me1) for the region surrounding rs7523831. The yellow vertical line indicates the position of the queried SNP.

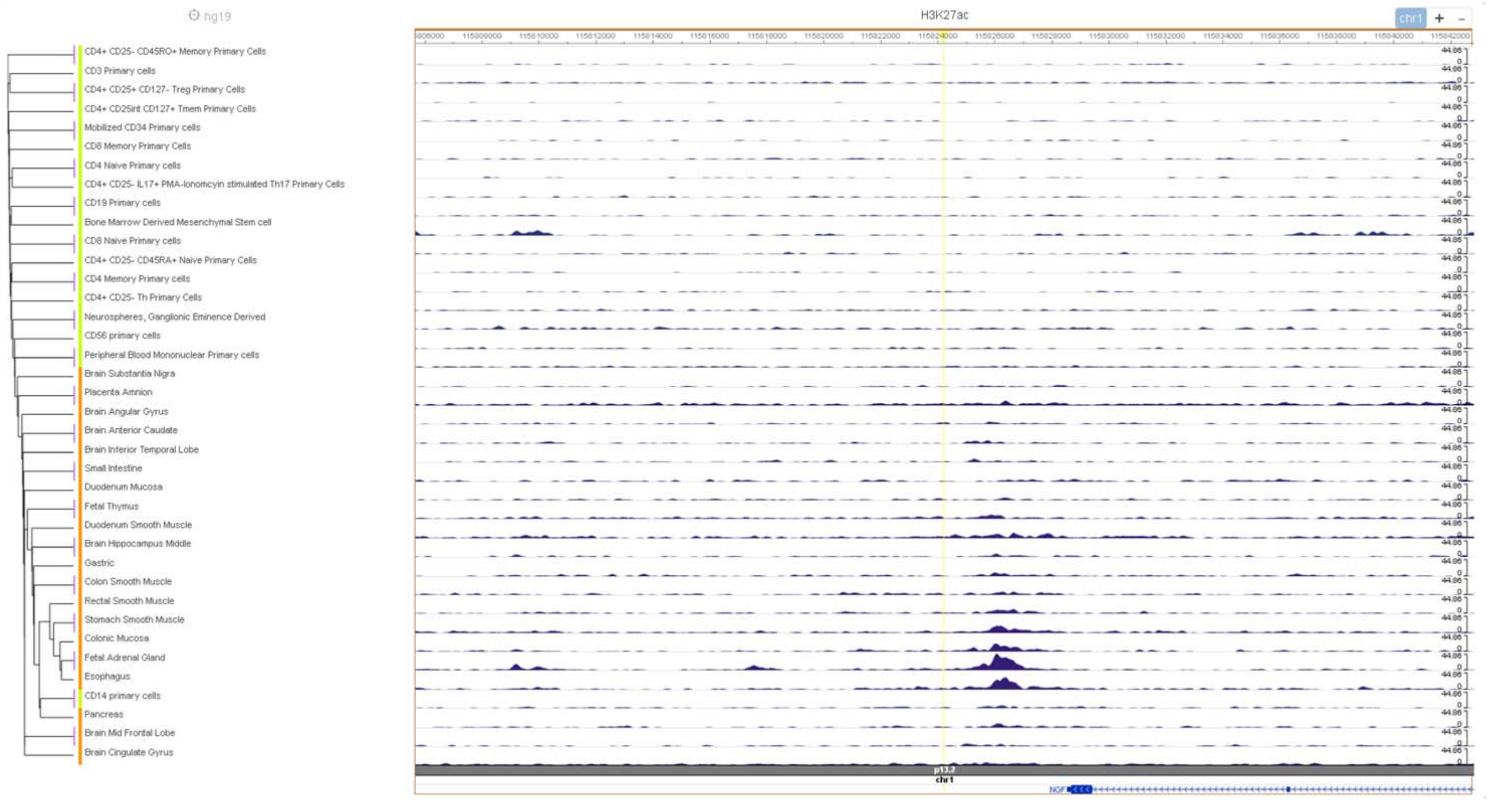

Supplementary Figure 6. Epigenomic annotation (H3K27ac) for the region surrounding rs7523831. The yellow vertical line indicates the position of the queried SNP.

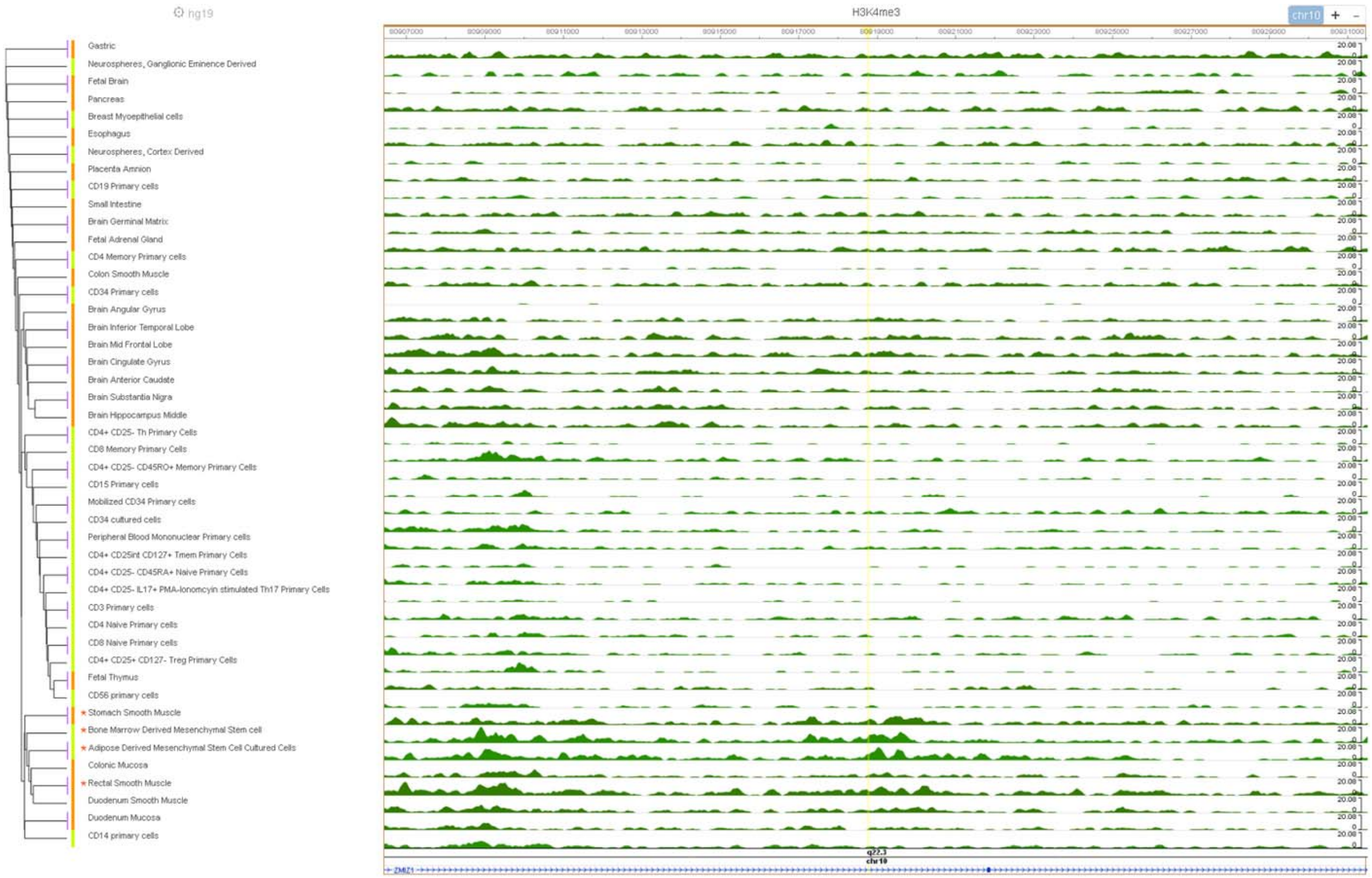

Supplementary Figure 7. Epigenomic annotation (H3K4me3) for the region surrounding rs76518691. The yellow vertical line indicates the position of the queried SNP.

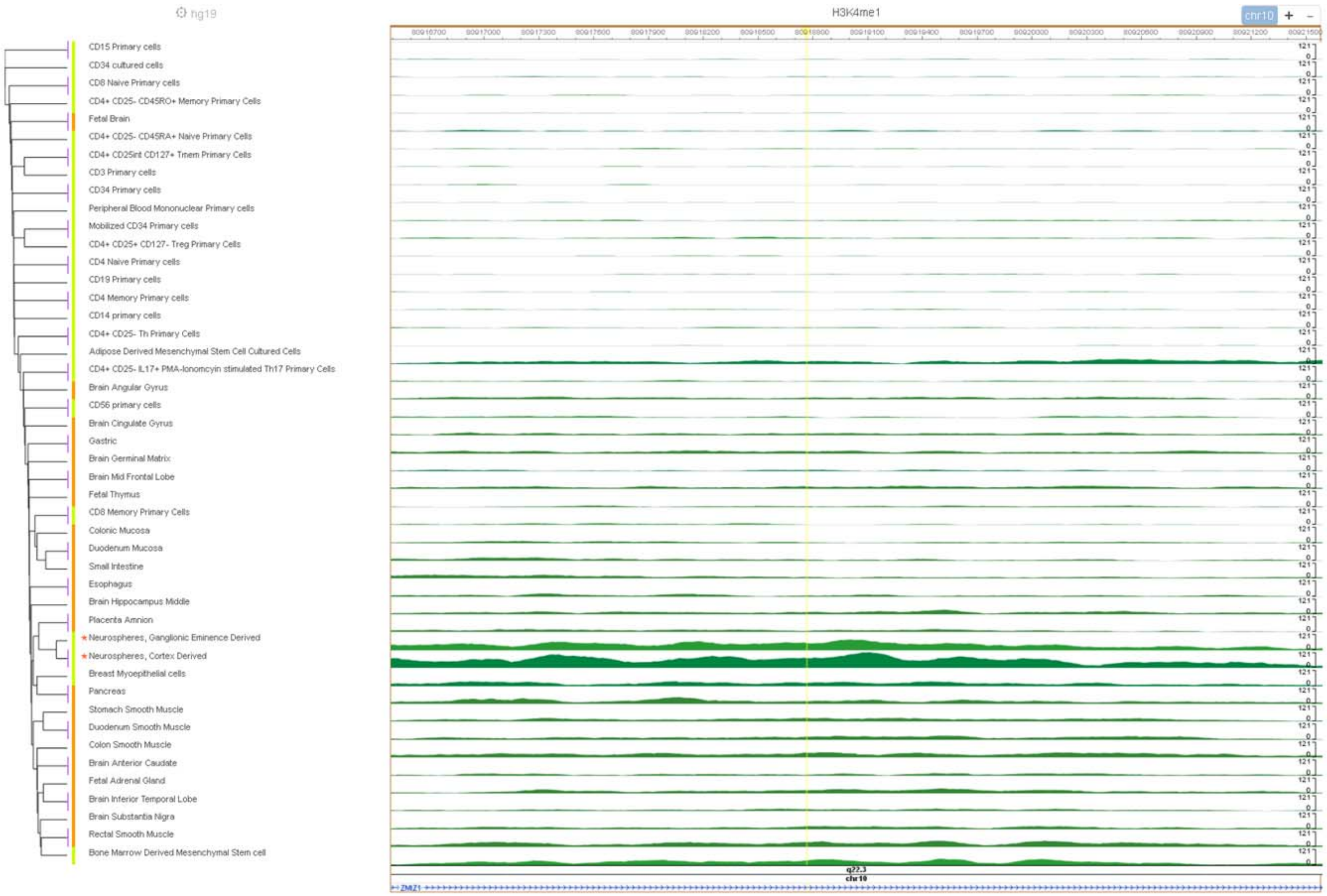

Supplementary Figure 8. Epigenomic annotation (H3K4me1) for the region surrounding rs76518691. The yellow vertical line indicates the position of the queried SNP.

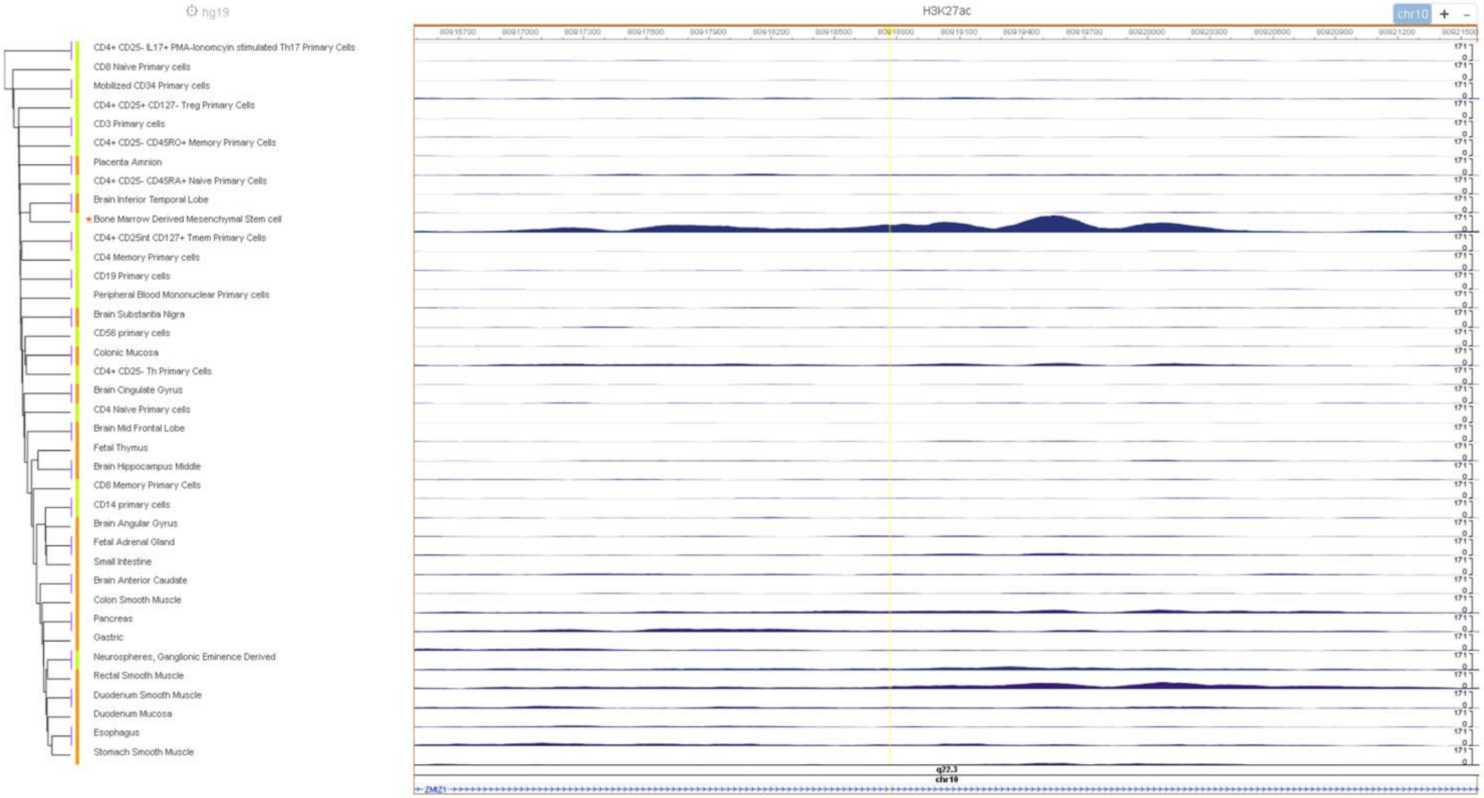

Supplementary Figure 9. Epigenomic annotation (H3K27ac) for the region surrounding rs76518691. The yellow vertical line indicates the position of the queried SNP.

Supplementary Table 1. Primary dysmenorrhea case and control sample sets (after QC)

	**Case**			**Control**		
	**N**	**Age, years mean (s.d.)**	**Age at menarche, years mean (s.d.)**	**N**	**Age, years mean (s.d.)**	**Age at menarche, years mean (s.d.)**
**Discovery stage**						
Data Set 1	709	18.4 (0.7)	13.0 (1.2)	1,279	18.3 (0.7)	13.2 (1.1)
Data Set 2	793	22.1 (4.3)	13.3 (1.3)	707	20.8 (3.7)	13.3 (1.2)
Data Set 3	902	19.0 (1.1)	13.6 (1.3)	934	19.0 (1.0)	13.9 (1.3)
**Replication stage**						
Data set 4	678	20.2 (3.4)	13.2 (1.2)	768	19.4 (3.0)	13.2 (1.1)
**Total**	**3,082**			**3,688**		

Supplementary Table 2. Sample and SNP levels quality control for GWAS cohorts.

	Sample QC (n)				PCA outlier (n)	SNP QC (n)			After QC
	Low quality	Inconsistent gender	Heterozygosity rate > mean ± 6 s.d	PI_HAT > 0.20		MAF < 3%	Call rate < 97%	HWE < 1e-6	
Data Set 1	12	4	5	13	24	351752	218961	593	645836
Data Set 2	19/11[a]	0	0	21	23	95245	78871	1386	1033567
Data Set 3	27/17[a]	7	3	30	41	80583	69212	1489	1057785

[a]The number here is for the Affymetrix Axiom® Genome-Wide CHB1/2 Array Plate Set, which includes two arrays (CHB1 and CHB2).

Supplementary Table 3. Association analysis results for the 16 SNPs in the Discovery, Replication stages and meta-analysis.

Chr.	SNP	Position (hg19)	A1/A2	Discovery stage			Replication stage			Meta-analysis			
				OR	SE	P	OR	SE	P	OR	SE	P	P_{het}
1	rs2294512	54,370,306	A/G	1.218	0.044	8.47E-06	0.894	0.079	0.156	1.131	0.039	1.40E-03	0.001
1	rs663045	108,743,059	C/G	1.195	0.040	9.46E-06	1.082	0.077	0.305	1.170	0.036	1.08E-05	0.255
1	rs7523831	115,824,192	C/G	0.837	0.040	6.94E-06	0.762	0.075	2.70E-04	0.820	0.035	1.36E-08	0.267
1	rs60441301	162,225,145	A/T	1.336	0.060	1.48E-06	1.115	0.123	0.376	1.290	0.054	2.42E-06	0.188
2	rs35205792	15,249,411	C/T	1.195	0.039	5.22E-06	0.993	0.076	0.925	1.149	0.035	6.20E-05	0.030
2	rs72829648	112,842,808	C/A	1.612	0.107	7.46E-06	0.965	0.215	0.869	1.456	0.095	8.21E-05	0.032
4	rs6551724	63,730,015	A/G	0.832	0.040	4.56E-06	0.924	0.073	0.275	0.853	0.035	5.65E-06	0.205
6	rs2027517	136,215,474	A/G	0.818	0.045	7.54E-06	1.049	0.085	0.576	0.864	0.040	2.14E-04	0.010
9	rs72741503	83,755,265	A/T	1.594	0.105	8.10E-06	1.037	0.185	0.843	1.437	0.091	6.80E-05	0.043
10	rs4750321	13,272,255	C/T	1.268	0.051	3.49E-06	1.039	0.086	0.654	1.204	0.044	2.48E-05	0.047
10	rs76518691	80,918,767	A/G	0.738	0.059	2.31E-07	0.696	0.114	1.55E-03	0.729	0.052	1.47E-09	0.653
11	rs3133223	120,605,232	A/G	1.317	0.060	5.02E-06	1.025	0.115	0.826	1.247	0.053	3.46E-05	0.053
15	rs11631919	63,250,239	C/G	0.628	0.104	7.62E-06	0.998	0.232	0.992	0.679	0.095	4.37E-05	0.068
16	rs79282103	5,928,452	A/G	1.356	0.068	7.08E-06	0.879	0.120	0.286	1.221	0.059	7.00E-04	0.002
19	rs6509898	55,222,106	A/G	1.527	0.091	3.34E-06	1.237	0.127	0.094	1.421	0.074	2.00E-06	0.178
22	rs1475944	47,307,287	T/C	0.811	0.045	4.33E-06	1.028	0.080	0.731	0.859	0.040	1.29E-04	0.010

Chr., chromosome; OR, odds ratio; SE, standard error; P_{het}, p-value for Cochrane's Q statistic.

Supplementary Table 4. Epigenomic analysis.

Chr.	Position	r2	D'	SNP	Ref	Alt	freq	GERP_cons	SiPhy_cons	Promoter histone marks	Enhancer histone marks	DNAse	Proteins bound	eQTL results	Motifs changed	RefSeq genes	Score
1	115265930	0.89	0.98	rs11583027	C	A	0.51	0	0		HRT, SKIN, BONE			2 eQTL results	ATF4,CEBPB,Pax-5	20kb 3' of NGF	6
1	115267227	0.89	-1	rs12759401	C	T	0.44	0	0	5 tissues	SKIN, ADRL, KID	MUS,SKIN		2 eQTL results	9 altered motifs	19kb 3' of NGF	5
1	115269118	0.97	1	rs34845081	AT	A	0.54	0	0						4 altered motifs	17kb 3' of NGF	6
1	115269758	0.97	1	rs11102914	A	G	0.54	0	0					3 eQTL results		16kb 3' of NGF	7
1	115270083	0.97	1	rs7542486	T	G	0.54	0	0					2 eQTL results	GR,Sox	16kb 3' of NGF	7
1	115271144	0.92	0.99	rs2057127	C	G	0.51	0	0					2 eQTL results	Pbx-1	15kb 3' of NGF	6
1	115271316	0.98	1	rs2207237	T	G	0.54	0	0					2 eQTL results	4 altered motifs	15kb 3' of NGF	6
1	115271679	0.98	1	rs5777229	TA	T	0.54	0	0						5 altered motifs	14kb 3' of NGF	6
1	115271862	0.98	1	rs2057126	C	T	0.54	0	0					2 eQTL results	5 altered motifs	14kb 3' of NGF	6

Chr.	Position	r2	D'	SNP	Ref	Alt	freq	GERP_cons	SiPhy_cons	Promoter histone marks	Enhancer histone marks	DNAse	Proteins bound	eQTL results	Motifs changed	RefSeq genes	Score
1	115271891	0.98	1	rs5021139	C	T	0.54	0	0						5 altered motifs	14kb 3' of NGF	7
1	115271928	0.98	1	rs2057125	G	T	0.54	0	0						5 altered motifs	14kb 3' of NGF	6
1	115272291	0.98	1	rs6678168	T	C	0.54	0	0						4 altered motifs	14kb 3' of NGF	6
1	115273844	0.98	1	rs2335399	T	G	0.54	0	0					2 eQTL results	MIF-1,RFX5	12kb 3' of NGF	7
1	115274600	0.97	0.99	rs12030576	G	T	0.53	0	0		FAT, ADRL			2 eQTL results	AP-1	11kb 3' of NGF	4
1	115276158	0.9	-1	rs2982742	G	T	0.44	1	1					2 eQTL results	Evi-1,Mef2	9.8kb 3' of NGF	7
1	115277977	0.98	1	rs11102915	T	C	0.54	0	0		FAT, STRM, KID				6 altered motifs	7.9kb 3' of NGF	4
1	115278464	0.9	-1	rs2068797	C	A	0.44	1	0		GI	KID		2 eQTL results	CIZ,HDAC2,RXRA	7.5kb 3' of NGF	4
1	115279844	0.92	0.99	rs6656381	T	A	0.51	0	0	FAT, SKIN	8 tissues			2 eQTL results	HNF4	6.1kb 3' of NGF	5
1	115280766	0.99	1	rs7523086	G	A	0.53	0	0	FAT, SKIN, LNG	7 tissues	SKIN		3 eQTL results	CEBPG	5.1kb 3' of NGF	7

Chr.	Position	r2	D'	SNP	Ref	Alt	freq	GERP_cons	SiPhy_cons	Promoter histone marks	Enhancer histone marks	DNAse	Proteins bound	eQTL results	Motifs changed	RefSeq genes	Score
**1**	**115281571**	**1**	**1**	**rs7523831**	**G**	**A,C**	**0.53**	**0**	**0**	**FAT, SKIN**	**5 tissues**			**2 eQTL results**		**4.3kb 3' of NGF**	**5**
1	115281777	0.97	0.99	rs7544256	A	G	0.53	1	1		5 tissues	OVRY			Mxi1,Myc	4.1kb 3' of NGF	5
1	115281894	1	1	rs7532754	C	T	0.53	0	0	SKIN	4 tissues	OVRY			STAT	4kb 3' of NGF	5
1	115282037	1	1	rs6678944	C	T	0.53	0	0		5 tissues					3.9kb 3' of NGF	5
1	115282910	0.98	1	rs6657049	G	A	0.54	0	0	FAT	8 tissues	16 tissues	PU1		6 altered motifs	3kb 3' of NGF	4
1	115283491	0.89	0.95	rs12075799	G	A	0.53	0	0	FAT	10 tissues	OVRY			5 altered motifs	2.4kb 3' of NGF	5
10	79146080	0.83	0.95	rs59674332	C	T	0.11	0	0	BLD	22 tissues	10 tissues			AP-1,Zbtb3	ZMIZ1	5
10	79149803	0.88	0.95	rs59791444	G	A	0.11	0	0	9 tissues	21 tissues	22 tissues			4 altered motifs	ZMIZ1	2b
**10**	**79159010**	**1**	**1**	**rs76518691**	**G**	**A**	**0.12**	**0**	**0**	**4 tissues**	**23 tissues**	**8 tissues**			**4 altered motifs**	**ZMIZ1**	**4**
10	79163152	1	1	rs78689605	G	T	0.12	0	0	CRVX	21 tissues	12 tissues	5 bound proteins		12 altered motifs	ZMIZ1	2b
10	79165529	1	1	rs74141795	G	C	0.12	0	0		17 tissues	7 tissues			NERF1a,Pou2f2	ZMIZ1	5

Chr.	Position	r2	D'	SNP	Ref	Alt	freq	GERP_cons	SiPhy_cons	Promoter histone marks	Enhancer histone marks	DNAse	Proteins bound	eQTL results	Motifs changed	RefSeq genes	Score
10	79172743	1	1	rs76285514	G	A	0.12	0	0		7 tissues				12 altered motifs	ZMIZ1	5

Data are shown for GWAS hits (marked in bold) and those in linkage disequilibrium (LD) with them (r2 > 0.8 in 1000 Genomes ASI phase 1 data) demonstrating evidence of histone marks, DNAse hypersensitivity sites or transcription factor occupancy using HaploReg v4 and RegulomeDB. Score: 2b, TF binding + any motif + DNase Footprint + DNase peak; 4, TF binding + DNase peak; 5, TF binding or DNase peak; 6, other; and 7, No Data.

Supplementary Table 5. NHGRI GWAS catalogue lookups for the primary dysmenorrhea associated regions.

Region	Position	SNP	PubMed ID	Journal	Title	Trait	Gene(s)	SNP-risk allele	Risk allele Freq	P value	OR or Beta	95% CI
1p13.2	115677946	rs12134493	23793025	Nat Genet	Genome-wide meta-analysis identifies new susceptibility loci for migraine.	Migraine	TSPAN2	rs12134493-A	0.12	5.00E-14	1.14	[1.10-1.18]
10q22.3	80819132	rs704017	24836286	Nat Genet	Large-scale genetic study in East Asians identifies six new loci associated with colorectal cancer risk.	Colorectal cancer	AS1, ZMIZ1	rs704017-G	0.32	2.00E-08	1.10	[1.06-1.13]
10q22.3	80841148	rs704010	23535729	Nat Genet	Large-scale genotyping identifies 41 new loci associated with breast cancer risk.	Breast cancer	ZMIZ1	rs704010-T	0.38	7.00E-22	1.08	[1.06-1.10]
10q22.3	80841148	rs704010	20453838	Nat Genet	Genome-wide association study identifies five new breast cancer susceptibility loci.	Breast cancer	ZMIZ1	rs704010-A	0.39	4.00E-09	1.07	[1.03-1.11]
10q22.3	80925577	rs7916441	20881960	Nature	Hundreds of variants clustered in genomic loci and biological pathways affect human height.	Height	PPIF	rs7916441-?	NR	6.00E-10		[NR]
10q22.3	80928793	rs1815314	25282103	Nat Genet	Defining the role of common variation in the genomic and biological architecture of adult human height.	Height	ZMIZ1	rs1815314-A	0.42	5.00E-14	0.02	[0.016-0.028] (unit decrease)
10q22.3	80931481	rs780151	23563607	Nat Genet	Genome-wide meta-analysis identifies 11 new loci for anthropometric traits and provides insights into genetic architecture.	Height	ZMIZ1	rs780151-G	0.57	2.00E-09	1.13	[NR]

Region	Position	SNP	PubMed ID	Journal	Title	Trait	Gene(s)	SNP-risk allele	Risk allele Freq	P value	OR or Beta	95% CI
10q22.3	80942631	rs12571751	24509480	Nat Genet	Genome-wide trans-ancestry meta-analysis provides insight into the genetic architecture of type 2 diabetes susceptibility.	Type 2 diabetes	ZMIZ1	rs12571751-A	0.51	2.00E-10	1.09	[1.06-1.13]
10q22.3	81032532	rs1250546	23128233	Nature	Host-microbe interactions have shaped the genetic architecture of inflammatory bowel disease.	Inflammatory bowel disease	intergenic	rs1250546-A	0.60	3.00E-18	1.10	[1.065-1.128]
10q22.3	81032885	rs1250544	22482804	Am J Hum Genet	Combined analysis of genome-wide association studies for Crohn disease and psoriasis identifies seven shared susceptibility loci.	Crohn's disease and psoriasis	ZMIZ1	rs1250544-G	NR	7.00E-14	1.16	[NR]
10q22.3	81058027	rs1250552	20190752	Nat Genet	Multiple common variants for celiac disease influencing immune gene expression.	Celiac disease	ZMIZ1	rs1250552-?	0.53	9.00E-10	1.12	[1.09-1.16]
10q22.3	81060317	rs1250550	21833088	Nature	Genetic risk and a primary role for cell-mediated immune mechanisms in multiple sclerosis.	Multiple sclerosis	ZMIZ1	rs1250550-A	NR	6.00E-09	1.10	[1.09-1.12]
10q22.3	81060317	rs1250550	21102463	Nat Genet	Genome-wide meta-analysis increases to 71 the number of confirmed Crohn's disease susceptibility loci.	Crohn's disease	ZMIZ1	rs1250550-G	0.67	1.00E-30	1.19	[1.15-1.23]
10q22.3	81060317	rs1250550	19915574	Nat Genet	Common variants at five new loci associated with early-onset inflammatory bowel disease.	Inflammatory bowel disease (early onset)	ZMIZ1	rs1250550-?	0.68	6.00E-09	1.16	[1.09-1.25]

Region	Position	SNP	PubMed ID	Journal	Title	Trait	Gene(s)	SNP-risk allele	Risk allele Freq	P value	OR or Beta	95% CI
10q22.3	81121696	rs2145998	20881960	Nature	Hundreds of variants clustered in genomic loci and biological pathways affect human height.	Height	PPIF	rs2145998-A	0.49	4.00E-13	0.03	[NR] (unit decrease)
10q22.3	81132829	rs1923367	25282103	Nat Genet	Defining the role of common variation in the genomic and biological architecture of adult human height.	Height	ZCCHC24	rs1923367-C	0.48	5.00E-24	0.03	[0.024-0.036] (unit decrease)

Supplementary Table 6. Gene-based p-values for the top 25 genes associated with primary dysmenorrhea in the discovery stage (using VEGAS2).

Chr	Gene	nSNPs	Start	Stop	Pvalue	TopSNP	TopSNP-pvalue
2	*IL1A*	27	113531491	113542971	2.60E-05	rs3783550	5.17E-05
5	*TRIM7*	8	180630121	180632293	3.40E-05	rs2770944	3.70E-05
2	*CKAP2L*	24	113495443	113522254	5.10E-05	rs6721044	2.54E-05
2	*IL36RN*	47	113816214	113822320	1.33E-04	rs2515404	3.15E-05
5	*CTC-338M12.4*	16	180673540	180684587	2.03E-04	rs2770966	1.49E-04
16	*LOC102724927*	8	3997625	4000445	2.39E-04	rs2283491	8.05E-05
14	*IRF2BPL*	6	77490885	77495042	2.52E-04	rs12897921	1.14E-04
5	*TRIM52*	8	180683385	180688119	2.84E-04	rs75954717	1.48E-04
1	*RHCE*	59	25688739	25747363	3.44E-04	rs586178	5.75E-05
5	*LOC257396*	11	52405671	52410956	3.72E-04	rs10072821	1.95E-04
22	*TBC1D22A*	1019	47158513	47571342	3.88E-04	rs1475944	4.33E-06
12	*ATP5G2*	39	54058943	54070512	4.20E-04	rs76321460	3.55E-04
1	*NBPF4*	7	108765962	108786703	4.22E-04	rs3870737	3.88E-04
5	*GNB2L1*	24	180663927	180670906	4.35E-04	rs2546404	8.08E-05
1	*OR2B11*	3	247614330	247615284	4.88E-04	rs12028142	7.00E-04
17	*CTC1*	32	8128138	8151413	4.91E-04	rs3027247	2.26E-04

Chr	Gene	nSNPs	Start	Stop	Pvalue	TopSNP	TopSNP-pvalue
2	*TMEM87B*	86	112812799	112876895	4.99E-04	rs72829648	7.46E-06
1	*SLC25A24*	150	108677343	108742980	5.27E-04	rs547364	1.79E-05
11	*MRPL16*	2	59573607	59578345	5.64E-04	rs139019298	1.10E-04
8	*DEPTOR*	513	120885894	121063157	5.69E-04	rs7388508	5.37E-04
8	*RRM2B*	49	103216728	103251346	5.80E-04	rs3735721	3.11E-04
17	*SCARF1*	16	1537151	1549083	6.06E-04	rs7220535	3.82E-04
5	*MOCS2*	35	52391508	52405602	6.07E-04	rs74550199	1.88E-04
14	*DPF3*	588	73086003	73360824	6.26E-04	rs148423638	3.42E-04
2	*ACMSD*	126	135596185	135659602	6.36E-04	rs6711390	4.63E-04

Supplementary Table 7. Pathway analysis of primary dysmenorrhea using the discovery GWAS data.

Database	Gene Set	Genes (mapped)	95th percentile enrichment cutoff			75th percentile enrichment cutoff		
			P	FDR	Enrichment Exp. (obs.)	P	FDR	Enrichment Exp. (obs.)
**GOTERM**	**interleukin-1 receptor binding**	**10 (9)**	**2.80E-05**	**0.0053**	**0 (5)**	0.0100	0.5637	2 (6)
**PANTHER_MOLECULAR_FUNCTION**	**Amylase**	**11 (6)**	**2.10E-03**	**0.0171**	**0 (3)**	0.0052	0.1110	2 (5)
**Ingenuity**	**BMP.Signaling.pathway**	**25 (24)**	**8.00E-04**	**0.0443**	**1 (6)**	0.0196	1.0000	6 (11)
**BIOCARTA**	**LEPTIN_PATHWAY**	**11 (11)**	**1.60E-03**	**0.0455**	**1 (4)**	0.2859	0.5083	3 (4)
**PANTHER_MOLECULAR_FUNCTION**	**Extracellular_matrix_glycoprotein**	**111 (87)**	0.8142	0.9841	4 (3)	**3.00E-04**	**0.0494**	**22 (37)**
GOTERM	decidualization	13 (13)	0.0003	0.0535	1 (5)	0.0817	0.7504	3 (6)
BIOCARTA	FCER1_PATHWAY	39 (36)	0.0001	0.0823	2 (8)	0.0036	0.2523	9 (17)
BIOCARTA	PYK2_PATHWAY	29 (28)	0.0026	0.0847	1 (6)	0.0722	0.3271	7 (11)
BIOCARTA	ERK_PATHWAY	28 (27)	0.0021	0.0909	1 (6)	0.0084	0.2160	7 (13)
BIOCARTA	NGF_PATHWAY	18 (17)	0.0092	0.1016	1 (4)	0.1066	0.3616	4 (7)
BIOCARTA	BCR_PATHWAY	35 (32)	0.0055	0.1020	2 (6)	0.0370	0.2846	8 (13)
BIOCARTA	TCR_PATHWAY	47 (43)	0.0056	0.1065	2 (7)	0.0495	0.3156	11 (16)
BIOCARTA	GLEEVEC_PATHWAY	23 (23)	0.0045	0.1095	1 (5)	0.0971	0.3584	6 (9)
BIOCARTA	BIOPEPTIDES_PATHWAY	43 (41)	0.0042	0.1101	2 (7)	0.0340	0.2895	10 (16)
BIOCARTA	CDK5_PATHWAY	11 (11)	0.0151	0.1111	1 (3)	0.0087	0.2388	3 (7)
BIOCARTA	MET_PATHWAY	37 (36)	0.0081	0.1130	2 (6)	0.0897	0.3629	9 (13)
BIOCARTA	TRKA_PATHWAY	12 (12)	0.0207	0.1247	1 (3)	0.0132	0.2328	3 (7)
BIOCARTA	AT1R_PATHWAY	34 (32)	0.0044	0.1279	2 (6)	0.1509	0.4373	8 (11)
BIOCARTA	HER2_PATHWAY	22 (22)	0.0231	0.1365	1 (4)	0.0302	0.2564	6 (10)
BIOCARTA	PDGF_PATHWAY	32 (31)	0.0179	0.1418	2 (5)	0.0635	0.3203	8 (12)
Ingenuity	Estrogen.Receptor.Signaling	31 (30)	0.0161	0.1487	2 (5)	0.1047	0.6995	8 (11)

Database	Gene Set	Genes (mapped)	95th percentile enrichment cutoff			75th percentile enrichment cutoff		
			P	FDR	Enrichment Exp. (obs.)	P	FDR	Enrichment Exp. (obs.)
BIOCARTA	IL1R_PATHWAY	33 (31)	0.0183	0.1498	2 (5)	0.1268	0.4145	8 (11)
BIOCARTA	RAC1_PATHWAY	23 (22)	0.0208	0.1527	1 (4)	0.2943	0.5430	6 (7)
Ingenuity	Ceramide.Signaling	16 (15)	0.0370	0.1622	1 (3)	0.0595	0.6646	4 (7)
BIOCARTA	EGF_PATHWAY	31 (30)	0.0181	0.1624	2 (5)	0.1030	0.3848	8 (11)
Ingenuity	Chemokine.Signaling	24 (23)	0.0255	0.1646	1 (4)	0.5287	0.8542	6 (6)
Ingenuity	NRF2-mediated.Oxidative.Stress.Response	51 (49)	0.0105	0.1648	2 (7)	0.1359	0.6221	12 (16)
Ingenuity	PXR.RXR.Activation	49 (48)	0.0092	0.1658	2 (7)	0.1216	0.6561	12 (16)
BIOCARTA	TOLL_PATHWAY	37 (33)	0.0232	0.1796	2 (5)	0.1037	0.3773	8 (12)
BIOCARTA	TPO_PATHWAY	24 (24)	0.0326	0.1932	1 (4)	0.1219	0.3897	6 (9)
PANTHER_MOLECULAR_FUNCTION	Adenylate_cyclase	10 (10)	0.0116	0.2075	1 (3)	0.0788	0.4176	3 (5)
BIOCARTA	FMLP_PATHWAY	37 (35)	0.0315	0.2110	2 (5)	0.0067	0.2487	9 (16)
BIOCARTA	CCR5_PATHWAY	18 (17)	0.0471	0.2123	1 (3)	0.6454	0.8146	4 (4)
BIOCARTA	LECTIN_PATHWAY	12 (9)	0.0725	0.2230	0 (2)	0.6957	0.8255	2 (2)
Ingenuity	Toll-like.Receptor.Signaling	33 (28)	0.0114	0.2253	1 (5)	0.0289	0.7743	7 (12)
BIOCARTA	INTEGRIN_PATHWAY	38 (37)	0.0356	0.2283	2 (5)	0.0621	0.3110	9 (14)
BIOCARTA	HCMV_PATHWAY	17 (17)	0.0505	0.2356	1 (3)	0.0388	0.3050	4 (8)
REACTOME	INNATE_IMMUNITY_SIGNALING	136 (95)	0.0193	0.2441	5 (10)	0.4165	0.9672	24 (25)
REACTOME	DOWN_STREAM_SIGNAL_TRANSDUCTION	35 (35)	0.0290	0.2484	2 (5)	0.1441	0.6913	9 (12)
BIOCARTA	CALCINEURIN_PATHWAY	19 (18)	0.0608	0.2505	1 (3)	0.0548	0.2916	5 (8)
REACTOME	PLATELET_ACTIVATION_TRIGGERS	59 (57)	0.0227	0.2560	3 (7)	0.0149	0.5771	14 (22)

Database	Gene Set	Genes (mapped)	95th percentile enrichment cutoff			75th percentile enrichment cutoff		
			P	FDR	Enrichment Exp. (obs.)	P	FDR	Enrichment Exp. (obs.)
REACTOME	CREB_PHOSPHORYLATION_THROUGH_THE_ACTIVATION_OF_CAMKII	16 (16)	0.0434	0.2580	1 (3)	0.0832	0.5372	4 (7)
REACTOME	PLC_GAMMA1_SIGNALLING	35 (35)	0.0084	0.2599	2 (6)	0.0785	0.5378	9 (13)
REACTOME	PKA_ACTIVATION	17 (17)	0.0475	0.2608	1 (3)	0.1062	0.5804	4 (7)
REACTOME	SIGNALLING_TO_RAS	26 (25)	0.0341	0.2617	1 (4)	0.0280	0.5533	6 (11)
REACTOME	ACTIVATED_AMPK_STIMULATES_FATTY_ACID_OXIDATION_IN_MUSCLE	17 (17)	0.0526	0.2640	1 (3)	0.4315	0.9692	4 (5)
REACTOME	PI3K_CASCADE	38 (37)	0.0362	0.2648	2 (5)	0.1869	0.7607	9 (12)
REACTOME	FRS2_MEDIATED_ACTIVATION	16 (16)	0.0399	0.2659	1 (3)	0.0247	0.5121	4 (8)
REACTOME	CAM_PATHWAY	26 (26)	0.0088	0.2689	1 (5)	0.0910	0.5781	7 (10)
REACTOME	DOWNSTREAM_SIGNALING_OF_ACTIVATED_FGFR	43 (42)	0.0161	0.2702	2 (6)	0.2264	0.8145	11 (13)
REACTOME	GAMMA_CARBOXYLATION_TRANSPORT_AND_AMINO_TERMINAL_CLEAVAGE_OF_PROTEINS	10 (8)	0.0578	0.2714	0 (2)	0.6295	0.9845	2 (2)
REACTOME	SIGNALING_BY_TGF_BETA	15 (15)	0.0351	0.2715	1 (3)	0.0537	0.5493	4 (7)
REACTOME	ENERGY_DEPENDENT_REGULATION_OF_MTOR_BY_LKB1_AMPK	17 (17)	0.0496	0.2718	1 (3)	0.4209	0.9593	4 (5)
REACTOME	MYD88_CASCADE	19 (15)	0.0353	0.2772	1 (3)	0.1513	0.6550	4 (6)
REACTOME	FORMATION_OF_PLATELET_PLUG	186 (173)	0.0142	0.2797	9 (16)	0.0389	0.5256	43 (54)
REACTOME	TRKA_SIGNALLING_FROM_THE_PLASMA_MEMBRANE	103 (101)	0.0103	0.2812	5 (11)	0.0180	0.5076	25 (35)
BIOCARTA	MAL_PATHWAY	19 (19)	0.0673	0.2835	1 (3)	0.1742	0.4315	5 (7)

Database	Gene Set	Genes (mapped)	95th percentile enrichment cutoff			75th percentile enrichment cutoff		
			P	FDR	Enrichment Exp. (obs.)	P	FDR	Enrichment Exp. (obs.)
Ingenuity	GM-CSF.Signaling	23 (21)	0.0843	0.2837	1 (3)	0.6338	0.8649	5 (5)
Ingenuity	IL-10.Signaling	25 (23)	0.1040	0.2848	1 (3)	0.0422	0.6755	6 (10)
REACTOME	CREB_PHOPHORYLATION_THROUGH_THE_ACTIVATION_OF_RAS	26 (24)	0.0314	0.2851	1 (4)	0.0216	0.5431	6 (11)
REACTOME	RAS_ACTIVATION_UOPN_CA2+_INFUX_THROUGH_NMDA_RECEPTOR	18 (18)	0.0576	0.2852	1 (3)	0.0588	0.5143	5 (8)
REACTOME	SOS_MEDIATED_SIGNALLING	13 (13)	0.0272	0.2864	1 (3)	0.0789	0.5507	3 (6)
REACTOME	VITAMIN_B5_(PANTOTHENATE)_METABOLISM	11 (11)	0.0150	0.2873	1 (3)	0.1186	0.5892	3 (5)
Ingenuity	Acute.Phase.Response.Signaling	75 (68)	0.0542	0.2877	3 (7)	0.8391	0.9258	17 (14)
REACTOME	GRB2_EVENTS_IN_EGFR_SIGNALING	13 (13)	0.0235	0.2878	1 (3)	0.0814	0.5281	3 (6)
REACTOME	GS_ALPHA_MEDIATED_EVENTS_IN_GLUCAGON_SIGNALLING	27 (27)	0.0480	0.2900	1 (4)	0.0566	0.5061	7 (11)
BIOCARTA	TGFB_PATHWAY	19 (19)	0.0691	0.2906	1 (3)	0.0292	0.2650	5 (9)
Ingenuity	Ephrin.Receptor.Signaling	36 (35)	0.0921	0.2910	2 (4)	0.1409	0.6475	9 (12)
REACTOME	REGULATION_OF_AMPK_ACTIVITY_VIA_LKB1	14 (14)	0.0319	0.2937	1 (3)	0.2611	0.8281	4 (5)
BIOCARTA	IGF1_PATHWAY	21 (20)	0.0753	0.2944	1 (3)	0.2075	0.4742	5 (7)
REACTOME	SHC_MEDIATED_SIGNALLING	12 (12)	0.0216	0.2948	1 (3)	0.0508	0.5237	3 (6)
REACTOME	SIGNALLING_TO_P38_VIA_RIT_AND_RIN	14 (14)	0.0313	0.2957	1 (3)	0.0384	0.5269	4 (7)
REACTOME	REGULATION_OF_RHEB_GTPASE_ACTIVITY_BY_AMPK	10 (10)	0.0101	0.2988	1 (3)	0.4810	0.9660	3 (3)

Database	Gene Set	Genes (mapped)	95th percentile enrichment cutoff			75th percentile enrichment cutoff		
			P	FDR	Enrichment Exp. (obs.)	P	FDR	Enrichment Exp. (obs.)
BIOCARTA	RAB_PATHWAY	12 (11)	0.1020	0.2998	1 (2)	0.2883	0.5158	3 (4)
BIOCARTA	NFKB_PATHWAY	23 (20)	0.0782	0.3008	1 (3)	0.3875	0.6368	5 (6)
REACTOME	G_ALPHA_Z_SIGNALLING_EVENTS	14 (14)	0.0292	0.3011	1 (3)	0.0369	0.4989	4 (7)
Ingenuity	Axonal.Guidance.Signaling	67 (63)	0.0906	0.3028	3 (6)	0.0869	0.7171	16 (21)
BIOCARTA	GPCR_PATHWAY	35 (32)	0.0794	0.3030	2 (4)	0.0162	0.2331	8 (14)
REACTOME	TOLL_LIKE_RECEPTOR_9_CASCADE	23 (19)	0.0143	0.3034	1 (4)	0.1782	0.7115	5 (7)
Ingenuity	PDGF.Signaling	23 (22)	0.0977	0.3037	1 (3)	0.6845	0.9051	6 (5)
Ingenuity	Natural.Killer.Cell.Signaling	26 (24)	0.1192	0.3050	1 (3)	0.3976	0.9211	6 (7)
BIOCARTA	BARR_MAPK_PATHWAY	12 (12)	0.1157	0.3053	1 (2)	0.1599	0.4285	3 (5)
BIOCARTA	IL6_PATHWAY	22 (21)	0.0851	0.3073	1 (3)	0.2486	0.5221	5 (7)
REACTOME	SHC_RELATED_EVENTS	14 (14)	0.0284	0.3082	1 (3)	0.0386	0.5526	4 (7)
BIOCARTA	INSULIN_PATHWAY	22 (21)	0.0881	0.3084	1 (3)	0.2579	0.5208	5 (7)
Panther	Coenzyme_A_biosynthesis	7 (7)	0.0435	0.3090	0 (2)	0.0119	0.3660	2 (5)
BIOCARTA	CCR3_PATHWAY	23 (21)	0.0811	0.3107	1 (3)	0.0193	0.2148	5 (10)
Ingenuity	cAMP-mediated.Signaling	11 (11)	0.1037	0.3109	1 (2)	0.5466	0.9345	3 (3)
REACTOME	EARLY_PHASE_OF_HIV_LIFE_CYCLE	13 (13)	0.0235	0.3117	1 (3)	0.1977	0.7199	3 (5)
BIOCARTA	CERAMIDE_PATHWAY	22 (21)	0.0867	0.3125	1 (3)	0.4280	0.6552	5 (6)
BIOCARTA	PARKIN_PATHWAY	12 (12)	0.1219	0.3156	1 (2)	0.3480	0.5614	3 (4)
KEGG	KEGG_LONG_TERM_POTENTIATION	70 (65)	0.0044	0.3176	3 (9)	0.1073	0.6776	16 (21)
GOTERM	HOPS complex	12 (12)	0.0019	0.3198	1 (4)	0.0530	0.7518	3 (6)
BIOCARTA	CACAM_PATHWAY	14 (13)	0.1286	0.3277	1 (2)	0.2076	0.4591	3 (5)
BIOCARTA	CXCR4_PATHWAY	24 (24)	0.1140	0.3288	1 (3)	0.0578	0.3243	6 (10)

Database	Gene Set	Genes (mapped)	95th percentile enrichment cutoff			75th percentile enrichment cutoff		
			P	FDR	Enrichment Exp. (obs.)	P	FDR	Enrichment Exp. (obs.)
BIOCARTA	IGF1R_PATHWAY	23 (23)	0.1058	0.3293	1 (3)	0.0411	0.2698	6 (10)
PANTHER_BIOLOGICAL_PROCESS	Skeletal_development	123 (112)	0.0089	0.3325	6 (12)	0.0529	0.5228	28 (36)
Ingenuity	EGF.Signaling	21 (20)	0.0732	0.3329	1 (3)	0.5913	0.8432	5 (5)
Ingenuity	Glucocorticoid.Receptor.Signaling	102 (95)	0.0479	0.3330	5 (9)	0.3300	0.9012	24 (26)
BIOCARTA	PGC1A_PATHWAY	24 (23)	0.1032	0.3355	1 (3)	0.0162	0.2988	6 (11)
BIOCARTA	PAR1_PATHWAY	37 (34)	0.0863	0.3382	2 (4)	0.0646	0.3023	9 (13)
REACTOME	ACTIVATED_TLR4_SIGNALLING	24 (20)	0.0699	0.3401	1 (3)	0.3794	0.9547	5 (6)
REACTOME	CALCITONIN_LIKE_LIGAND_RECEPTORS	10 (10)	0.0863	0.3412	1 (2)	0.7612	0.9561	3 (2)
REACTOME	POST_NMDA_RECEPTOR_ACTIVATION_EVENTS	32 (30)	0.0631	0.3413	2 (4)	0.0203	0.4845	8 (13)
REACTOME	ADENYLATE_CYCLASE_ACTIVATING_PATHWAY	11 (11)	0.0173	0.3419	1 (3)	0.1186	0.5938	3 (5)
REACTOME	INTEGRIN_CELL_SURFACE_INTERACTIONS	81 (77)	0.0350	0.3419	4 (8)	0.0849	0.5951	19 (25)
REACTOME	SIGNAL_AMPLIFICATION	31 (30)	0.0606	0.3464	2 (4)	0.1059	0.5751	8 (11)
REACTOME	CELL_SURFACE_INTERACTIONS_AT_THE_VASCULAR_WALL	94 (92)	0.0379	0.3503	5 (9)	0.1950	0.7728	23 (27)
REACTOME	NCAM_SIGNALING_FOR_NEURITE_OUT_GROWTH	69 (66)	0.0460	0.3514	3 (7)	0.0123	0.6533	17 (25)
Ingenuity	TGF-beta.Signaling	30 (29)	0.0556	0.3518	1 (4)	0.1737	0.6364	7 (10)
REACTOME	TOLL_RECEPTOR_CASCADES	86 (78)	0.0396	0.3532	4 (8)	0.2132	0.8220	20 (23)
REACTOME	SIGNALING_BY_PDGF	64 (62)	0.0028	0.3534	3 (9)	0.0107	0.7761	16 (24)
REACTOME	SHCMEDIATED_CASCADE	21 (20)	0.0810	0.3535	1 (3)	0.3823	0.9524	5 (6)

Database	Gene Set	Genes (mapped)	95th percentile enrichment cutoff			75th percentile enrichment cutoff		
			P	FDR	Enrichment Exp. (obs.)	P	FDR	Enrichment Exp. (obs.)
Panther	Endothelin_signaling_pathway	19 (19)	0.0117	0.3552	1 (4)	0.3330	0.8497	5 (6)
REACTOME	G_ALPHA_S_SIGNALLING_EVENTS	126 (123)	0.0443	0.3610	6 (11)	0.2790	0.9208	31 (34)
BIOCARTA	WNT_PATHWAY	26 (26)	0.1424	0.3676	1 (3)	0.0927	0.3590	7 (10)
Panther	Circadian_clock_system	9 (9)	0.0768	0.3688	0 (2)	0.1650	0.7215	2 (4)
REACTOME	IONOTROPIC_ACTIVITY_OF_KAINATE_RECEPTORS	12 (11)	0.1035	0.3696	1 (2)	0.2833	0.8284	3 (4)
BIOCARTA	NFAT_PATHWAY	54 (53)	0.1236	0.3698	3 (5)	0.0131	0.2403	13 (21)
Ingenuity	IL-6.Signaling	29 (27)	0.1479	0.3701	1 (3)	0.5227	0.8892	7 (7)
BIOCARTA	COMP_PATHWAY	19 (15)	0.1642	0.3705	1 (2)	0.9219	0.9414	4 (2)
BIOCARTA	BARRESTIN_SRC_PATHWAY	15 (15)	0.1763	0.3718	1 (2)	0.1498	0.4221	4 (6)
Panther	Muscarinic_acetylcholine_receptor_1_and_3_signaling_pathway	8 (8)	0.0576	0.3721	0 (2)	0.3174	0.7414	2 (3)
REACTOME	PLATELET_ACTIVATION	167 (154)	0.0441	0.3727	8 (13)	0.0485	0.5667	39 (48)
REACTOME	SPHINGOLIPID_METABOLISM	32 (32)	0.0739	0.3742	2 (4)	0.0786	0.5439	8 (12)
Panther	Endogenous_cannabinoid_signaling	2 (2)	0.0966	0.3752	0 (1)	0.4293	0.7552	1 (1)
BIOCARTA	VIP_PATHWAY	27 (26)	0.1414	0.3757	1 (3)	0.0175	0.2462	7 (12)
BIOCARTA	PITX2_PATHWAY	15 (15)	0.1713	0.3764	1 (2)	0.7583	0.8727	4 (3)
PANTHER_BIOLOGICAL_PROCESS	MHCI-mediated_immunity	22 (9)	0.0719	0.3771	0 (2)	0.0494	0.3716	2 (5)
BIOCARTA	GSK3_PATHWAY	27 (26)	0.1365	0.3789	1 (3)	0.1818	0.4468	7 (9)
BIOCARTA	IL3_PATHWAY	15 (14)	0.1537	0.3796	1 (2)	0.4824	0.6682	4 (4)
PANTHER_BIOLOGICAL_PROCESS	Receptor_mediated_endocytosis	111 (97)	0.0104	0.3799	5 (11)	0.0299	0.3928	24 (33)
REACTOME	OPIOID_SIGNALLING	83 (82)	0.0536	0.3800	4 (8)	0.0393	0.5155	21 (28)

Database	Gene Set	Genes (mapped)	95th percentile enrichment cutoff			75th percentile enrichment cutoff		
			P	FDR	Enrichment Exp. (obs.)	P	FDR	Enrichment Exp. (obs.)
BIOCARTA	MYOSIN_PATHWAY	31 (28)	0.1655	0.3807	1 (3)	0.3999	0.6359	7 (8)
BIOCARTA	CREB_PATHWAY	27 (27)	0.1477	0.3812	1 (3)	0.0206	0.2314	7 (12)
PANTHER_BIOLOGICAL_PROCESS	Fatty_acid_metabolism	120 (94)	0.0069	0.3820	5 (11)	0.2332	0.7944	24 (27)
BIOCARTA	PPARA_PATHWAY	58 (55)	0.1396	0.3824	3 (5)	0.2900	0.5705	14 (16)
REACTOME	THROMBOXANE_SIGNALLING_THROUGH_TP_RECEPTOR	23 (22)	0.0991	0.3832	1 (3)	0.1648	0.7073	6 (8)
REACTOME	AMINE_DERIVED_HORMONES	13 (12)	0.1155	0.3834	1 (2)	0.5994	0.9823	3 (3)
REACTOME	SIGNALING_BY_BMP	23 (22)	0.0972	0.3837	1 (3)	0.0805	0.5428	6 (9)
BIOCARTA	IL7_PATHWAY	17 (16)	0.1847	0.3847	1 (2)	0.3666	0.5962	4 (5)
REACTOME	LYSOSOME_VESICLE_BIOGENESIS	24 (22)	0.0947	0.3849	1 (3)	0.0735	0.5423	6 (9)
BIOCARTA	GH_PATHWAY	28 (28)	0.1658	0.3855	1 (3)	0.2552	0.5060	7 (9)
REACTOME	PROSTANOID_HORMONES	11 (11)	0.1059	0.3856	1 (2)	0.5476	0.9450	3 (3)
REACTOME	PHASE_1_FUNCTIONALIZATION	15 (12)	0.1145	0.3860	1 (2)	0.3469	0.9174	3 (4)
REACTOME	GLUCAGON_SIGNALING_IN_METABOLIC_REGULATION	34 (34)	0.0831	0.3861	2 (4)	0.0605	0.5278	9 (13)
REACTOME	DEPOLARIZATION_OF_THE_PRESYNAPTIC_TERMINAL_TRIGGERS_THE_OPENING_OF_CALCIUM_CHANNELS	12 (12)	0.1220	0.3877	1 (2)	0.0513	0.5267	3 (6)
REACTOME	SIGNALLING_TO_ERKS	34 (33)	0.0791	0.3878	2 (4)	0.0479	0.4999	8 (13)
REACTOME	COLLAGEN_MEDIATED_ACTIVATION_CASCADE	23 (22)	0.0960	0.3887	1 (3)	0.0737	0.5329	6 (9)
REACTOME	ACTIVATION_OF_NMDA_RECEPTOR_UPON_GLUTAMATE_BINDING_AND_POS	36 (33)	0.0832	0.3894	2 (4)	0.0451	0.5355	8 (13)

Database	Gene Set	Genes (mapped)	95th percentile enrichment cutoff			75th percentile enrichment cutoff		
			P	FDR	Enrichment Exp. (obs.)	P	FDR	Enrichment Exp. (obs.)
	TSYNAPTIC_EVENTS							
PANTHER_BIOLOGICAL_PROCESS	rRNA_metabolism	66 (60)	0.0288	0.3906	3 (7)	0.3160	0.7225	15 (17)
PANTHER_BIOLOGICAL_PROCESS	Gluconeogenesis	17 (16)	0.0401	0.3908	1 (3)	0.5917	0.8722	4 (4)
PANTHER_BIOLOGICAL_PROCESS	Steroid_hormone-mediated_signaling	52 (37)	0.0370	0.3930	2 (5)	0.3067	0.7398	9 (11)
PANTHER_BIOLOGICAL_PROCESS	MHCII-mediated_immunity	34 (10)	0.0114	0.3948	1 (3)	0.2258	0.7355	3 (4)
PANTHER_BIOLOGICAL_PROCESS	Glycogen_metabolism	52 (39)	0.0455	0.3966	2 (5)	0.0095	0.3387	10 (17)
PANTHER_BIOLOGICAL_PROCESS	Other_mRNA_transcription	21 (18)	0.0597	0.3991	1 (3)	0.0048	0.3768	5 (10)
BIOCARTA	SPRY_PATHWAY	18 (17)	0.2071	0.3992	1 (2)	0.0413	0.2980	4 (8)
BIOCARTA	IL10_PATHWAY	17 (16)	0.1940	0.4002	1 (2)	0.1880	0.4341	4 (6)
BIOCARTA	NDKDYNAMIN_PATHWAY	19 (17)	0.2077	0.4003	1 (2)	0.4318	0.6359	4 (5)
BIOCARTA	NKCELLS_PATHWAY	20 (17)	0.2046	0.4033	1 (2)	0.1002	0.3620	4 (7)
REACTOME	GLYCOGEN_BREAKDOWN_GLYCOGEN OLYSIS	16 (13)	0.1348	0.4040	1 (2)	0.6704	0.9629	3 (3)
Panther	TGF-beta_signaling_pathway	64 (60)	0.0315	0.4044	3 (7)	0.1532	0.7250	15 (19)
REACTOME	PLATELET_ADHESION_TO_EXPOSED_C OLLAGEN	14 (13)	0.1285	0.4051	1 (2)	0.0810	0.5332	3 (6)
GOTERM	bone mineralization	15 (14)	0.0041	0.4066	1 (4)	0.0355	0.7043	4 (7)
PANTHER_BIOLOGICAL_PROCESS	Protein_targeting	113 (96)	0.0216	0.4082	5 (10)	0.3531	0.7616	24 (26)
REACTOME	ADP_SIGNALLING_THROUGH_P2Y_PUR INOCEPTOR_1	25 (24)	0.1118	0.4121	1 (3)	0.2322	0.7989	6 (8)
REACTOME	TOLL_LIKE_RECEPTOR_4_CASCADE	28 (24)	0.1151	0.4130	1 (3)	0.3927	0.9613	6 (7)
BIOCARTA	ARF_PATHWAY	17 (17)	0.2158	0.4153	1 (2)	0.2390	0.5117	4 (6)

Database	Gene Set	Genes (mapped)	95th percentile enrichment cutoff			75th percentile enrichment cutoff		
			P	FDR	Enrichment Exp. (obs.)	P	FDR	Enrichment Exp. (obs.)
REACTOME	METABOLISM_OF_NITRIC_OXIDE	13 (13)	0.1345	0.4155	1 (2)	0.4130	0.9542	3 (4)
GOTERM	odontogenesis	16 (15)	0.0044	0.4160	1 (4)	0.0040	1.0000	4 (9)
BIOCARTA	EPO_PATHWAY	19 (18)	0.2271	0.4193	1 (2)	0.6953	0.8484	5 (4)
GOTERM	response to vitamin D	15 (15)	0.0058	0.4193	1 (4)	0.1446	0.8254	4 (6)
REACTOME	IRS_RELATED_EVENTS	79 (78)	0.0013	0.4201	4 (11)	0.0988	0.5854	20 (25)
PANTHER_BIOLOGICAL_PROCESS	Endoderm_development	12 (11)	0.1000	0.4225	1 (2)	0.1108	0.5047	3 (5)
BIOCARTA	PTEN_PATHWAY	18 (18)	0.2247	0.4233	1 (2)	0.2766	0.5055	5 (6)
PANTHER_BIOLOGICAL_PROCESS	Translational_regulation	88 (57)	0.0619	0.4267	3 (6)	0.3401	0.7369	14 (16)
PANTHER_BIOLOGICAL_PROCESS	Protein_targeting_and_localization	104 (56)	0.0234	0.4291	3 (7)	0.0272	0.3678	14 (21)
GOTERM	negative regulation of osteoblast differentiation	17 (16)	0.0069	0.4320	1 (4)	0.0256	0.6505	4 (8)
PANTHER_BIOLOGICAL_PROCESS	Other_neuronal_activity	136 (122)	0.0421	0.4330	6 (11)	0.2581	0.7593	31 (34)
REACTOME	TRANSMISSION_ACROSS_CHEMICAL_SYNAPSES	130 (122)	0.0848	0.4341	6 (10)	0.0497	0.5372	31 (39)
Panther	p53_pathway_by_glucose_deprivation	11 (10)	0.0131	0.4367	1 (3)	0.2216	0.6782	3 (4)
PANTHER_BIOLOGICAL_PROCESS	Complement-mediated_immunity	56 (45)	0.0674	0.4370	2 (5)	0.9053	0.9801	11 (8)
REACTOME	POST_TRANSLATIONAL_PROTEIN_MODIFICATION	40 (37)	0.1055	0.4371	2 (4)	0.4473	0.9636	9 (10)
PANTHER_BIOLOGICAL_PROCESS	Metabolism_of_cyclic_nucleotides	45 (43)	0.0666	0.4389	2 (5)	0.0259	0.3988	11 (17)
BIOCARTA	CARM_ER_PATHWAY	35 (32)	0.2145	0.4418	2 (3)	0.1590	0.4398	8 (11)
PANTHER_BIOLOGICAL_PROCESS	Other_homeostasis_activities	69 (57)	0.0071	0.4461	3 (8)	0.0171	0.3443	14 (22)
REACTOME	SEMA3A_PAK_DEPENDENT_AXON_REP	15 (14)	0.1518	0.4499	1 (2)	0.4777	0.9816	4 (4)

Database	Gene Set	Genes (mapped)	95th percentile enrichment cutoff			75th percentile enrichment cutoff		
			P	FDR	Enrichment Exp. (obs.)	P	FDR	Enrichment Exp. (obs.)
	ULSION							
PANTHER_BIOLOGICAL_PROCESS	Regulated_exocytosis	48 (45)	0.0761	0.4521	2 (5)	0.0756	0.5235	11 (16)
PANTHER_BIOLOGICAL_PROCESS	Vitamin/cofactor_transport	48 (35)	0.0887	0.4526	2 (4)	0.0158	0.3597	9 (15)
BIOCARTA	MTA3_PATHWAY	19 (19)	0.2451	0.4543	1 (2)	0.7293	0.8551	5 (4)
BIOCARTA	GCR_PATHWAY	20 (19)	0.2410	0.4586	1 (2)	0.5290	0.7119	5 (5)
PANTHER_BIOLOGICAL_PROCESS	Muscle_contraction	198 (166)	0.0381	0.4622	8 (14)	0.2293	0.7696	42 (46)
Panther	P53_pathway_feedback_loops_1	4 (4)	0.1907	0.4624	0 (1)	0.0490	0.4997	1 (3)
PANTHER_BIOLOGICAL_PROCESS	Miscellaneous	140 (119)	0.0732	0.4660	6 (10)	0.1139	0.5699	30 (36)
Ingenuity	VDR.RXR.Activation	63 (62)	0.2007	0.4667	3 (5)	0.4857	0.9045	16 (16)
BIOCARTA	IL2_PATHWAY	22 (20)	0.2681	0.4685	1 (2)	0.3879	0.6377	5 (6)
REACTOME	CELLEXTRACELLULAR_MATRIX_INTERACTIONS	16 (14)	0.1622	0.4687	1 (2)	0.1241	0.5867	4 (6)
REACTOME	NEURORANSMITTER_RECEPTOR_BINDING_AND_DOWNSTREAM_TRANSMISSION_IN_THE_POSTSYNAPTIC_CELL	84 (79)	0.1040	0.4699	4 (7)	0.0136	0.6409	20 (29)
GOTERM	cellular component movement	101 (94)	0.0006	0.4738	5 (13)	0.1738	0.8966	24 (28)
Ingenuity	G-Protein.Coupled.Receptor.Signaling	19 (19)	0.2462	0.4765	1 (2)	0.5269	0.8724	5 (5)
REACTOME	G_ALPHA_I_SIGNALLING_EVENTS	177 (160)	0.0988	0.4769	8 (12)	0.4537	0.9452	40 (41)
Ingenuity	SAPK.JNK.Signaling	32 (30)	0.1897	0.4802	2 (3)	0.4891	0.9384	8 (8)
REACTOME	GRB2_SOS_PROVIDES_LINKAGE_TO_MAPK_SIGNALING_FOR_INTERGRINS	15 (15)	0.1753	0.4812	1 (2)	0.5335	0.9448	4 (4)
REACTOME	CRMPS_IN_SEMA3A_SIGNALING	16 (15)	0.1681	0.4815	1 (2)	0.0176	0.8681	4 (8)

Database	Gene Set	Genes (mapped)	95th percentile enrichment cutoff			75th percentile enrichment cutoff		
			P	FDR	Enrichment Exp. (obs.)	P	FDR	Enrichment Exp. (obs.)
Ingenuity	Antigen.Presentation.Pathway	11 (7)	0.3031	0.4840	0 (1)	0.2398	0.5769	2 (3)
Ingenuity	PPAR.Signaling	19 (18)	0.2304	0.4840	1 (2)	0.2845	0.7630	5 (6)
GOTERM	negative regulation of BMP signaling pathway	20 (20)	0.0023	0.4852	1 (5)	0.0013	0.8285	5 (12)
Panther	Muscarinic_acetylcholine_receptor_2_and_4_signaling_pathway	5 (5)	0.2219	0.4853	0 (1)	0.7634	0.8434	1 (1)
Panther	Ubiquitin_proteasome_pathway	52 (47)	0.0821	0.4886	2 (5)	0.7745	0.8940	12 (10)
PANTHER_BIOLOGICAL_PROCESS	Phosphate_metabolism	91 (77)	0.0921	0.4886	4 (7)	0.6702	0.8876	19 (18)
PANTHER_BIOLOGICAL_PROCESS	Muscle_development	143 (121)	0.0808	0.4902	6 (10)	0.6325	0.8898	30 (29)
Panther	Thyrotropin-releasing_hormone_receptor_signaling_pathway	11 (11)	0.1019	0.4943	1 (2)	0.5392	0.8493	3 (3)
REACTOME	MTOR_SIGNALLING	27 (27)	0.1450	0.4960	1 (3)	0.8396	0.9584	7 (5)
Panther	Toll_receptor_signaling_pathway	30 (26)	0.1379	0.4983	1 (3)	0.4870	0.8650	7 (7)
Ingenuity	FGF.Signaling	18 (18)	0.2314	0.4989	1 (2)	0.7010	0.8902	5 (4)
Panther	VEGF_signaling_pathway	14 (14)	0.1525	0.4992	1 (2)	0.7227	0.8237	4 (3)
KEGG	KEGG_GNRH_SIGNALING_PATHWAY	101 (94)	0.0201	0.5029	5 (10)	0.0288	0.4736	24 (32)
Panther	General_transcription_by_RNA_polymerase_I	12 (12)	0.1182	0.5077	1 (2)	0.0133	0.3156	3 (7)
KEGG	KEGG_MELANOGENESIS	102 (95)	0.0026	0.5124	5 (12)	0.0340	0.4940	24 (32)
REACTOME	NCAM1_INTERACTIONS	44 (41)	0.1486	0.5212	2 (4)	0.0315	0.4733	10 (16)
BIOCARTA	SPPA_PATHWAY	22 (22)	0.2980	0.5319	1 (2)	0.1590	0.4393	6 (8)
PANTHER_MOLECULAR_FUNCTION	Other_enzyme_inhibitor	16 (13)	0.1336	0.5341	1 (2)	0.0797	0.4072	3 (6)

Database	Gene Set	Genes (mapped)	95th percentile enrichment cutoff			75th percentile enrichment cutoff		
			P	FDR	Enrichment Exp. (obs.)	P	FDR	Enrichment Exp. (obs.)
PANTHER_MOLECULAR_FUNCTION	Cell_adhesion_molecule	99 (85)	0.0611	0.5448	4 (8)	0.0011	0.0722	21 (34)
PANTHER_MOLECULAR_FUNCTION	Major_histocompatibility_complex_antigen	46 (12)	0.1190	0.5465	1 (2)	0.0504	0.3499	3 (6)
PANTHER_MOLECULAR_FUNCTION	Ion_channel	140 (119)	0.0706	0.5509	6 (10)	0.0336	0.3720	30 (39)
PANTHER_MOLECULAR_FUNCTION	Nuclear_hormone_receptor	47 (45)	0.0733	0.5644	2 (5)	0.0196	0.2829	11 (18)
BIOCARTA	PLATELETAPP_PATHWAY	14 (10)	0.3980	0.5828	1 (1)	0.0818	0.3221	3 (5)
KEGG	KEGG_VEGF_SIGNALING_PATHWAY	76 (73)	0.1594	0.5947	4 (6)	0.0267	0.4945	18 (26)
GOTERM	antigen processing and presentation of peptide antigen via MHC class I	11 (5)	0.0238	0.5950	0 (2)	0.0159	0.4244	1 (4)
BIOCARTA	RACCYCD_PATHWAY	26 (25)	0.3579	0.6174	1 (2)	0.0679	0.3002	6 (10)
BIOCARTA	EDG1_PATHWAY	27 (27)	0.3972	0.6234	1 (2)	0.0236	0.2280	7 (12)
PANTHER_MOLECULAR_FUNCTION	Lyase	16 (16)	0.1915	0.6307	1 (2)	0.0833	0.3907	4 (7)
REACTOME	AXON_GUIDANCE	161 (155)	0.1545	0.6308	8 (11)	0.0252	0.4947	39 (50)
PANTHER_BIOLOGICAL_PROCESS	Receptor_protein_serine/threonine_kinase_signaling_pathway	40 (35)	0.2512	0.6514	2 (3)	0.0067	0.3537	9 (16)
BIOCARTA	HDAC_PATHWAY	30 (29)	0.4251	0.6688	1 (2)	0.0388	0.2763	7 (12)
BIOCARTA	CDMAC_PATHWAY	16 (15)	0.5279	0.6761	1 (1)	0.1489	0.4277	4 (6)
BIOCARTA	BCELLSURVIVAL_PATHWAY	16 (15)	0.5353	0.6823	1 (1)	0.1370	0.4032	4 (6)
BIOCARTA	CDC42RAC_PATHWAY	16 (15)	0.5334	0.6852	1 (1)	0.0567	0.3043	4 (7)
PANTHER_BIOLOGICAL_PROCESS	mRNA_transcription_termination	10 (7)	0.3006	0.6870	0 (1)	0.0710	0.3782	2 (4)
PANTHER_MOLECULAR_FUNCTION	Transcription_factor	198 (128)	0.1949	0.7015	6 (9)	0.0283	0.3466	32 (42)
BIOCARTA	LONGEVITY_PATHWAY	15 (15)	0.5416	0.7028	1 (1)	0.1508	0.4160	4 (6)

Database	Gene Set	Genes (mapped)	95th percentile enrichment cutoff			75th percentile enrichment cutoff		
			P	FDR	Enrichment Exp. (obs.)	P	FDR	Enrichment Exp. (obs.)
PANTHER_MOLECULAR_FUNCTION	Cysteine_protease	125 (94)	0.1936	0.7214	5 (7)	0.0508	0.3897	24 (31)
PANTHER_BIOLOGICAL_PROCESS	Other_signal_transduction	71 (58)	0.3354	0.7224	3 (4)	0.0388	0.3916	15 (21)
PANTHER_MOLECULAR_FUNCTION	mRNA_processing_factor	27 (21)	0.2766	0.7296	1 (2)	0.0192	0.2801	5 (10)
GOTERM	synaptic transmission	166 (156)	0.0938	0.7298	8 (12)	0.0031	0.4423	39 (54)
PANTHER_MOLECULAR_FUNCTION	Phosphodiesterase	39 (36)	0.2691	0.7401	2 (3)	0.0032	0.0793	9 (17)
PANTHER_MOLECULAR_FUNCTION	Actin_binding_cytoskeletal_protein	106 (76)	0.1735	0.7422	4 (6)	0.0011	0.0933	19 (31)
BIOCARTA	CYTOKINE_PATHWAY	21 (18)	0.6075	0.7457	1 (1)	0.0547	0.2973	5 (8)
BIOCARTA	MEF2D_PATHWAY	21 (18)	0.5990	0.7490	1 (1)	0.0527	0.2975	5 (8)
PANTHER_MOLECULAR_FUNCTION	Extracellular_matrix_linker_protein	23 (21)	0.0874	0.7507	1 (3)	0.0234	0.2702	5 (10)
BIOCARTA	ERK5_PATHWAY	18 (18)	0.6073	0.7522	1 (1)	0.1421	0.4208	5 (7)
BIOCARTA	IGF1MTOR_PATHWAY	20 (19)	0.6265	0.7574	1 (1)	0.1729	0.4261	5 (7)
REACTOME	INORGANIC_CATION_ANION_SLC_TRANSPORTERS	94 (89)	0.2860	0.7582	4 (6)	0.0241	0.4821	22 (31)
BIOCARTA	DC_PATHWAY	22 (20)	0.6441	0.7596	1 (1)	0.2238	0.4911	5 (7)
BIOCARTA	STATHMIN_PATHWAY	19 (19)	0.6235	0.7604	1 (1)	0.1709	0.4340	5 (7)
PANTHER_BIOLOGICAL_PROCESS	Regulation_of_carbohydrate_metabolism	13 (12)	0.4621	0.7681	1 (1)	0.0148	0.2804	3 (7)
GOTERM	positive regulation of interleukin-8 production	10 (9)	0.0665	0.7728	0 (2)	0.0096	0.4716	2 (6)
BIOCARTA	NTHI_PATHWAY	24 (23)	0.6893	0.7887	1 (1)	0.0962	0.3508	6 (9)
GOTERM	hippocampus development	17 (16)	0.0434	0.7893	1 (3)	0.0081	0.4435	4 (9)
BIOCARTA	INFLAM_PATHWAY	29 (24)	0.7096	0.8003	1 (1)	0.1274	0.3859	6 (9)

Database	Gene Set	Genes (mapped)	95th percentile enrichment cutoff			75th percentile enrichment cutoff		
			P	FDR	Enrichment Exp. (obs.)	P	FDR	Enrichment Exp. (obs.)
GOTERM	positive regulation of protein kinase activity	16 (16)	0.0475	0.8095	1 (3)	0.0076	0.4675	4 (9)
GOTERM	3',5'-cyclic-AMP phosphodiesterase activity	9 (9)	0.0729	0.8096	0 (2)	0.0093	0.4890	2 (6)
REACTOME	AMINO_ACID_TRANSPORT_ACROSS_THE_PLASMA_MEMBRANE	31 (28)	0.4096	0.8152	1 (2)	0.0008	0.2643	7 (15)
KEGG	KEGG_LEISHMANIA_INFECTION	72 (54)	0.5125	0.8217	3 (3)	0.0085	0.3514	14 (22)
BIOCARTA	FAS_PATHWAY	30 (30)	0.7881	0.8259	2 (1)	0.1960	0.4779	8 (10)
KEGG	KEGG_ALDOSTERONE_REGULATED_SODIUM_REABSORPTION	42 (40)	0.5924	0.8350	2 (2)	0.0014	0.2005	10 (19)
BIOCARTA	IL5_PATHWAY	10 (8)	1.0000	0.8552	0 (0)	0.1157	0.3543	2 (4)
BIOCARTA	VITCB_PATHWAY	11 (8)	1.0000	0.8578	0 (0)	0.0049	0.2849	2 (6)
BIOCARTA	ACE2_PATHWAY	13 (8)	1.0000	0.8591	0 (0)	0.1165	0.3482	2 (4)
BIOCARTA	IL4_PATHWAY	11 (10)	1.0000	0.8643	1 (0)	0.2229	0.4566	3 (4)
PANTHER_MOLECULAR_FUNCTION	Interleukin	34 (29)	0.0570	0.8660	1 (4)	0.0158	0.2435	7 (13)
BIOCARTA	CBL_PATHWAY	13 (12)	1.0000	0.8841	1 (0)	0.1568	0.4248	3 (5)
PANTHER_BIOLOGICAL_PROCESS	MAPKKK_cascade	181 (158)	0.5381	0.8944	8 (8)	0.0169	0.3646	40 (52)
BIOCARTA	STEM_PATHWAY	15 (15)	1.0000	0.9018	1 (0)	0.1480	0.4227	4 (6)
PANTHER_MOLECULAR_FUNCTION	ATP-binding_cassette_(ABC)_transporter	46 (39)	0.5875	0.9023	2 (2)	0.0091	0.1776	10 (17)
BIOCARTA	TH1TH2_PATHWAY	19 (16)	1.0000	0.9047	1 (0)	0.1900	0.4289	4 (6)
BIOCARTA	GATA3_PATHWAY	16 (15)	1.0000	0.9047	1 (0)	0.0176	0.2692	4 (8)
PANTHER_MOLECULAR_FUNCTION	Serine_protease_inhibitor	76 (63)	0.3878	0.9066	3 (4)	0.0490	0.4368	16 (22)

Database	Gene Set	Genes (mapped)	95th percentile enrichment cutoff			75th percentile enrichment cutoff		
			**P**	**FDR**	**Enrichment Exp. (obs.)**	**P**	**FDR**	**Enrichment Exp. (obs.)**
PANTHER_MOLECULAR_FUNCTION	GABA_receptor	20 (16)	0.5623	0.9096	1 (1)	0.0814	0.3954	4 (7)
PANTHER_MOLECULAR_FUNCTION	Cation_transporter	118 (105)	0.0152	0.9231	5 (11)	0.0509	0.3878	26 (34)
PANTHER_MOLECULAR_FUNCTION	Other_enzyme_activator	17 (16)	0.5658	0.9304	1 (1)	0.0791	0.3812	4 (7)
PANTHER_MOLECULAR_FUNCTION	Guanyl-nucleotide_exchange_factor	138 (124)	0.4269	0.9326	6 (7)	0.0174	0.2906	31 (42)
PANTHER_BIOLOGICAL_PROCESS	Determination_of_dorsal/ventral_axis	16 (14)	1.0000	0.9565	1 (0)	0.1071	0.4893	4 (6)
PANTHER_MOLECULAR_FUNCTION	Other_nucleic_acid_binding	41 (29)	0.7729	0.9605	1 (1)	0.0769	0.4349	7 (11)
PANTHER_BIOLOGICAL_PROCESS	Amino_acid_catabolism	50 (40)	1.0000	0.9809	2 (0)	0.0250	0.3604	10 (16)
Panther	Salvage_pyrimidine_ribonucleotides	6 (5)	1.0000	0.9873	0 (0)	0.1044	0.4643	1 (3)
PANTHER_MOLECULAR_FUNCTION	Miscellaneous_function	87 (69)	0.8673	0.9925	3 (2)	0.0247	0.3282	17 (25)
PANTHER_MOLECULAR_FUNCTION	Interleukin_receptor	40 (27)	1.0000	0.9952	1 (0)	0.0539	0.4041	7 (11)
PANTHER_MOLECULAR_FUNCTION	Tyrosine_protein_kinase_receptor	77 (69)	1.0000	1.0000	3 (0)	0.0412	0.4173	17 (24)

The pathways that reached study-wise statistical significance (FDR <0.05) were marked in bold, and those with FDR>0.5 are not shown. Genes denotes number of genes in pathway (number of genes successfully mapped by MAGENTA). Enrichment denotes expected number of genes at enrichment threshold (observed number of genes).

Supplementary Table 8. Functional Partitioning of SNP Heritability of primary dysmenorrhea.

Group	h_g^2	SE	h_g^2 %	SE. h_g^2 %	SNP %	Enrichment
**Coding**	0.0549	0.0406	24.0%	17.8%	1.5%	16.3
**UTR**	0.0000	0.0230	0.0%	10.0%	0.6%	0.0
**Promoter**	0.0047	0.0384	2.0%	16.8%	2.2%	0.9
**DHS**	0.1393	0.1067	60.9%	46.7%	16.1%	3.8
**Intron**	0.0000	0.0605	0.0%	26.5%	31.0%	0.0
**Intergenic**	0.0298	0.0611	13.0%	26.7%	48.6%	0.3
**Sum**	**0.2288**	**0.0587**				

Supplementary Table 9. GWAS identified endometriosis associated loci.

Region	Gene(s)	Chr.	Position	SNP	Risk Allele	Risk Allele Freq	P Value	OR	95% CI	Pubmed ID
1p36.12	*LOC100289113, ZBTB40, WNT4*	1	22450487	rs2235529	rs2235529-A	0.153	3.00E-09	1.3	[1.19-1.41]	23472165
1p36.12	*WNT4*	1	22490724	rs7521902	rs7521902-A	0.238	3.00E-11	1.19	[1.13-1.25]	23104006
2p25.1	*GREB1*	2	11727507	rs13394619	rs13394619-G	0.521	6.00E-09	1.15	[1.09-1.20]	23104006
6p22.3	NR	6	19785588	rs7739264	rs7739264-T	0.515	4.00E-10	1.17	[1.11-1.23]	23104006
7p15.2	intergenic	7	25901639	rs12700667	rs12700667-A	0.744	4.00E-09	1.18	[1.11-1.25]	23104006
7p15.2	intergenic	7	25901639	rs12700667	rs12700667-A	0.74	1.00E-09	1.2	[1.13-1.27]	21151130
9p21.3	*CDKN2BAS*	9	22115105	rs10965235	rs10965235-C	0.802	6.00E-12	1.44	[1.30-1.59]	20601957
9p21.3	*CDKN2B-ASI, ARF, CDKN2A, CDKN2B*	9	22169700	rs1537377	rs1537377-C	0.401	2.00E-09	1.15	[1.10-1.21]	23104006
12q22	*VEZT*	12	95711876	rs10859871	rs10859871-C	0.295	5.00E-13	1.2	[1.14-1.26]	23104006

Supplementary Table 10. Association analysis of the endometriosis associated SNPs with primary dysmenorrhea.

Chr.	SNP	Position	A1/A2	OR	SE	P_value
1	rs2235529	22450487	A/G	0.984	0.039	0.682
1	rs7521902	22490724	A/C	0.974	0.040	0.516
2	rs13394619	11727507	A/G	0.938	0.039	0.100
6	rs7739264	19785588	T/C	1.048	0.046	0.303
7	rs12700667	25901639	A/G	0.942	0.050	0.237
9	rs10965235	22115105	A/C	0.906	0.050	0.051
9	rs1537377	22169700	C/T	0.899	0.043	0.013
12	rs10859871	95711876	C/A	0.940	0.045	0.167
